# Integrated cardio-behavioral responses to threat define defensive states

**DOI:** 10.1038/s41593-022-01252-w

**Published:** 2023-02-09

**Authors:** Jérémy Signoret-Genest, Nina Schukraft, Sara L. Reis, Dennis Segebarth, Karl Deisseroth, Philip Tovote

**Affiliations:** 1 Institute of Clinical Neurobiology, University Hospital Wuerzburg, Wuerzburg, Germany; 2 Center for Mental Health, University Hospital Wuerzburg, Wuerzburg, Germany; 3 Howard Hughes Medical Institute and Departments of Bioengineering and Psychiatry, Stanford University, Stanford, CA USA

**Keywords:** Limbic system, Neural circuits

## Abstract

Fear and anxiety are brain states that evolved to mediate defensive responses to threats. The defense reaction includes multiple interacting behavioral, autonomic and endocrine adjustments, but their integrative nature is poorly understood. In particular, although threat has been associated with various cardiac changes, there is no clear consensus regarding the relevance of these changes for the integrated defense reaction. Here we identify rapid microstates that are associated with specific behaviors and heart rate dynamics, which are affected by long-lasting macrostates and reflect context-dependent threat levels. In addition, we demonstrate that one of the most commonly used defensive behavioral responses—freezing as measured by immobility—is part of an integrated cardio-behavioral microstate mediated by *Chx10*^+^ neurons in the periaqueductal gray. Our framework for systematic integration of cardiac and behavioral readouts presents the basis for a better understanding of complex neural defensive states and their associated systemic functions.

## Abstract (web summary)

Tovote and colleagues present an analytical framework that integrates threat-evoked inter-related behavioral and cardiac adjustments and allows identification of short- and long-lasting defensive states and their midbrain neuronal mediators.

## Main

The defense reaction in response to a threat—a central element of human fear or anxiety—encompasses multiple behavioral, autonomic and endocrine adjustments, which are controlled and integrated by neural circuits^1^. Based almost exclusively on behavioral responses, a unifying, across-species concept describes the dynamic nature of defensive responses as ‘states’^2–4^. However, due to this focus on behavioral dynamics alone, a comprehensive understanding of defensive states and the integrated nature of their individual components remain incomplete. Yet, this is critical to causally link the known variety of neuronal states to their systemic readouts, such as behavior^5^. Brain circuitry involving the midbrain periaqueductal gray (PAG) has long been suggested to have a major role in mediating defensive states by integrating behavioral and cardiac components^6–9^, but a lack of integrated analyses has prevented clarification of precise circuit mechanisms.

Although investigations of defensive states have predominantly focused on threat-induced behavioral changes^10,11^, several studies have also addressed ‘autonomic’ adaptations to threats, in particular changes in heart rate (HR)^12–15^. Similar to behavioral responses, HR is considered the product of multiple internal processes that are sensitive to threats. In contrast to relatively robust defensive behaviors elicited under tightly controlled experimental conditions, studies on defensive autonomic responses have yielded complex, paradoxical observations and sometimes seemingly contradictory findings. Indeed, under threat conditions, both deceleration (bradycardia) as well acceleration (tachycardia) of HR have been reported^8,13,16–23^. Possibly because of these heterogeneous results, many studies either ignore cardiac readouts as quantifiable measures for defensive states or equate specific HR responses directly with fear.

In this study, we apply new analyses to a large dataset of concomitant behavioral, HR and thermal measures during various behavioral paradigms in freely moving mice. Based on these analyses, we identify and define transient microstates and their interaction with longer-lasting macrostates to explain both tachycardic and bradycardic defensive responses and associated behavioral patterns. We reveal that integrated defense states are cue- and context-dependent and show that HR indices precisely identify defensive state transitions and contextual threat levels. Furthermore, using optogenetic perturbational approaches, we show that *Chx10*^+^ neurons in the midbrain PAG have a particular ‘state generator’ role. Overall, we introduce a new framework for the characterization of integrated cardio-behavioral defensive states that may serve as the basis for a comprehensive understanding of complex neuronal mechanisms underlying aversive emotions such as fear and anxiety.

## HR changes determine defensive microstates and macrostates

**Fig. 1:**
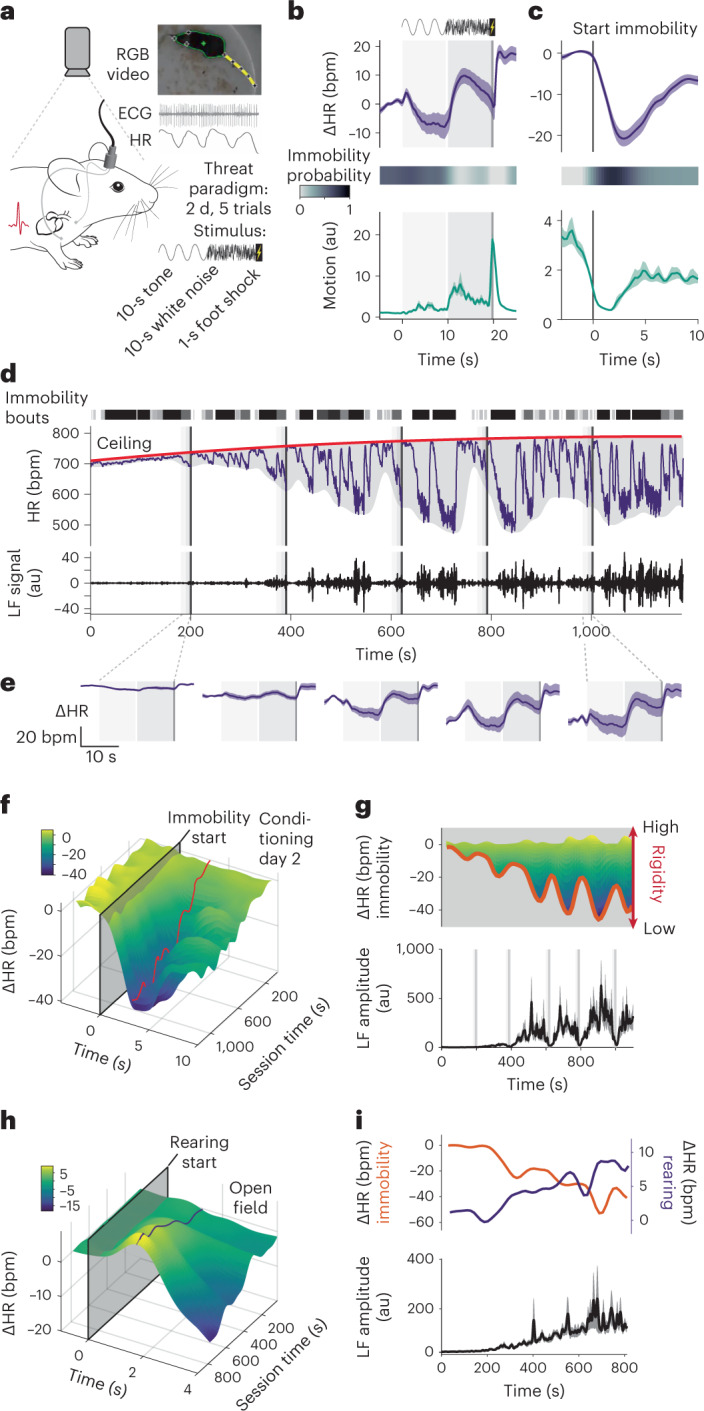
HR changes reflect the interaction between defensive microstates and macrostates. **a**, Electrocardiograms were recorded from video-tracked freely moving mice to concomitantly study HR readouts and behavior in a 2-d fear conditioning paradigm for which a compound stimulus (tone then white noise) is paired with a shock. **b**,**c**, PSTHs showing the mean ΔHR ± s.e.m. (top), immobility probability (middle) and average motion ± s.e.m. (bottom) to the 5 CS–US pairings during the second day of conditioning (**b**, *n* = 33 mice) or spontaneous immobility episodes (**c**, *n* = 33 mice); in both cases, immobility was associated with pronounced bradycardia. **d**, Example trace from a single mouse for a conditioning day 2 recording. Shaded rectangles indicate CS–US pairings (five in total). Raw HR (middle) illustrates how bradycardic events are clearly correlated with individual immobility bouts (dashed lines on top). It also shows that HR progressively increases from the beginning of the recording, following a latent maximum, the ceiling (red curve). The overall amplitude of the HR changes increases over time, as visualized by the smoothed envelope in gray. Finally, HR presents oscillations in the LF range, variability which is obvious during bradycardic episodes (middle curve), or after isolating the corresponding signal from the raw HR (bottom curve). **e**, PSTHs showing the mean ΔHR ± s.e.m. for each of the five individual CS–US pairings (*n* = 33 mice), on which the progressive increase in amplitude is clearly visible. **f**, Three-dimensional representation showing the increase in amplitude of the immobility-associated HR changes in function of the recording time for conditioning day 2. The red line represents the lowest value for each time point. **g**, Top, side view from **f** to better show the dynamics. The amplitude of immobility-associated bradycardia over time (red curve, same as **f**) reflects rigidity. Bottom, average quantification of the LF amplitude ± s.e.m. (*n* = 33 mice), developing over time and appearing to be correlated to rigidity. **h**, Three-dimensional representation showing the increase in rearing-associated HR changes along time in the OF (*n* = 23 mice). **i**, Top, curves of immobility- and rearing-associated HR changes during OF recordings; bottom, average LF amplitude ± s.e.m. during the same recordings (*n* = 23 mice). Mouse image in **a** is adapted from SciDraw.

**Extended Data Fig. 1:**
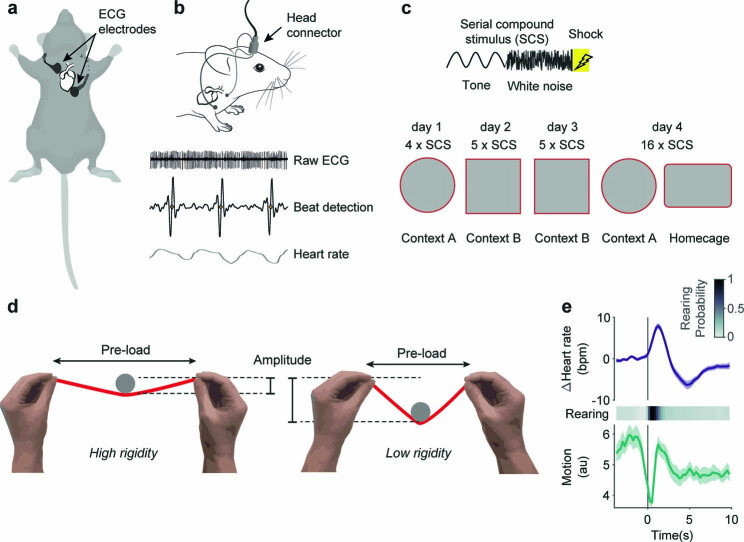
Approaches for cardio-behavioral characterization of defensive states. **a**, Scheme showing the electrodes’ subcutaneous placement for ECG recordings. **b**, Electrodes are soldered to a connector in order to acquire the signal, from which heart beats are extracted to process HR. **c**, Description of the conditioned flight paradigm. Top, the serial compound stimulus consists in pure tone beeps followed by white noise bursts, and shock for conditioning days. Bottom, contexts and number of presentations for the successive days. **d**, Analogy to explain rigidity: rigidity would be like the tension of a stretched rubber band, opposing deflection. For a same state (the same object placed on the rubber), for instance immobility, if rigidity (the rubber tension) is high, it will restrict the associated decrease in HR (the deflection of the rubber band). **e**, PSTHs showing the mean HR ± s.e.m. (top) and behavioral responses (middle and bottom) accompanying rearing bouts (OF, n = 23 mice).

As a prerequisite for understanding integrated defensive states, we exposed freely moving mice implanted with electrocardiogram (ECG) electrodes (Fig. 1a and Extended Data Fig. 1a,b) to a recently developed conditioned flight paradigm in which a serial compound stimulus (SCS), an auditory cue (pure tone followed by white noise), is paired with a mild electrical footshock, eliciting rapid switches in the behavioral state^24^ (Extended Data Fig. 1c). Mice exhibited stimulus-specific combinations of HR and behavioral responses as follows: the pure tone period was characterized by immobility and a decrease in HR, whereas white noise was accompanied by interspersed flight/immobility and an increase in HR (Fig. 1b and Supplementary Video 1). HR during spontaneous, non-CS-evoked immobility episodes decreased likewise (Fig. 1c and Supplementary Video 1), suggesting the existence of a stereotypical transient cardio-behavioral microstate characterized by immobility and HR decrease, that is bradycardia. However, the amplitude of immobility-associated bradycardia increased progressively throughout the conditioning session (Fig. 1d,e).

How can we explain such a progressive change in the microstate level? Crucially, the state change affected the cardiac component of both spontaneous and CS-evoked immobility-bradycardia microstate (Fig. 1d–f) and was paralleled by low-frequency (LF) oscillations of HR. These fluctuations of the inter-heartbeat interval are a murine equivalent to the LF band of human HR variability, which has been associated with emotional states^25^ (Fig. 1d, bottom panel). This led us to hypothesize that one or several underlying processes were interfering with HR dynamics at a more global level, and in particular with the expression of the cardiovascular component of the defensive microstate. Overall, HR evolved within the boundaries of a smooth envelope, where minimum values mainly corresponded to the HR decreases associated with immobility episodes, and maximum values never exceeded a monotonic curve (Fig. 1d, top panel). Because those changes operated at extended timescales when compared with short-lasting, for example, cue-induced immobility bouts, we refer to them as dynamic ‘macrostates’. To capture the slowly shifting upper boundary of HR, we defined the ‘ceiling’ macrostate, operationalized by latent maximum HR value, therefore progressively increasing in our conditions. In contrast, we attribute changes in the lower HR boundary to the ‘rigidity’ macrostate, which constrains the range of values that HR can reach by opposing changes (Extended Data Fig. 1d). This is reflected by the initial small amplitude of LF HR variability fluctuations and immobility-associated bradycardia, which increases over time (Fig. 1e–g). Important to note, while ceiling increased, rigidity decreased across the session, which was reflected by an increase in bradycardia amplitude.

Beyond threat conditioning, we wondered if our macrostates were (1) also present in other contexts, and therefore dependent on general underlying processes and (2) affected other microstates in a similar fashion. Using the commonly used open field (OF) test, we found another microstate, in which rearing was associated with a transient increase in HR (Fig. 1h and Extended Data Fig. 1e). The amplitude of that tachycardia increased over the course of the recording and was correlated with amplitude increases in LF HR variability fluctuations and immobility-associated bradycardia (Fig. 1i), similar to the progressive changes in immobility-associated bradycardia during conditioning. This confirms that the rigidity macrostate reflects a general mechanism affecting cardiac responses under threat conditions.

Taken together, these findings suggest that HR dynamics do not simply follow changes in behavioral activity but reflect integrated defensive microstates with behavioral and autonomic components. Furthermore, we identified slow macrostate changes that exert strong influences on moment-to-moment microstates, such as those induced by threatening cues.

## Inter-related dynamics of defensive states

**Fig. 2:**
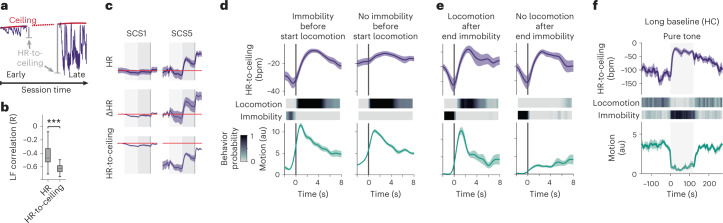
Defense state dynamics determine cardiac responses. **a**, HR-to-ceiling is the Δ between the HR value at time *t* and the ceiling. Because of decreasing rigidity, the values appear greater further from the beginning of the recording. **b**, LF amplitude was only poorly correlated to raw HR but well correlated with HR-to-ceiling (*n* = 33 mice; two-tailed Mann–Whitney *U* test; see Supplementary Table 1 for details). **c**, Comparison of raw HR (top), ΔHR (middle) and HR-to-ceiling (bottom) average responses (mean ± s.e.m., *n* = 33 mice) for SCS1 (left) and SCS5 (right). The red line shows the baseline values before SCS1 to help comparison. While the dynamics are globally similar, raw HR and ΔHR suffer from higher variability and encourage improper interpretations because their values do not capture an absolute level. **d**, PSTHs showing mean HR-to-ceiling ± s.e.m. (top), locomotion and immobility probabilities (middle) and average motion ± s.e.m. (bottom) built around locomotion bouts start (*n* = 23 mice, OF), split into two groups depending on whether immobility was present shortly before the locomotion bout (left panel) or not (right panel; see also heat maps in the middle). In both cases, there is an increase in motion consistent with the locomotion (bottom), and the HR-to-ceiling value during locomotion is similar (top). However, there is a clear increase in HR-to-ceiling only when transitioning from immobility to locomotion, not otherwise. **e**, PSTHs built around immobility bouts end (*n* = 23 mice, OF), split into two groups depending on whether immobility was directly followed by locomotion (left panel) or not (right panel; see also heat maps in the middle). HR-to-ceiling values increased in both cases, showing that the relevant state change is exiting immobility, and not engaging in locomotion. **f**, CS retrieval after a long baseline in the homecage (HC) led to crisp immobility (probability heat map (middle) and mean motion curve ± s.e.m. (bottom); *n* = 8 mice), which was accompanied by a pronounced increase in HR (average HR-to-ceiling ± s.e.m., top). This highlights the dependency of the cardiac response not only on the stimulus and behavior but also on the conditions and context at a specific time point. In the box whisker plot, boxes span the first to third quartiles (Q1 to Q3), horizontal line denotes the median and whiskers show the most extreme data points. ***P < 0.001.

**Extended Data Fig. 2:**
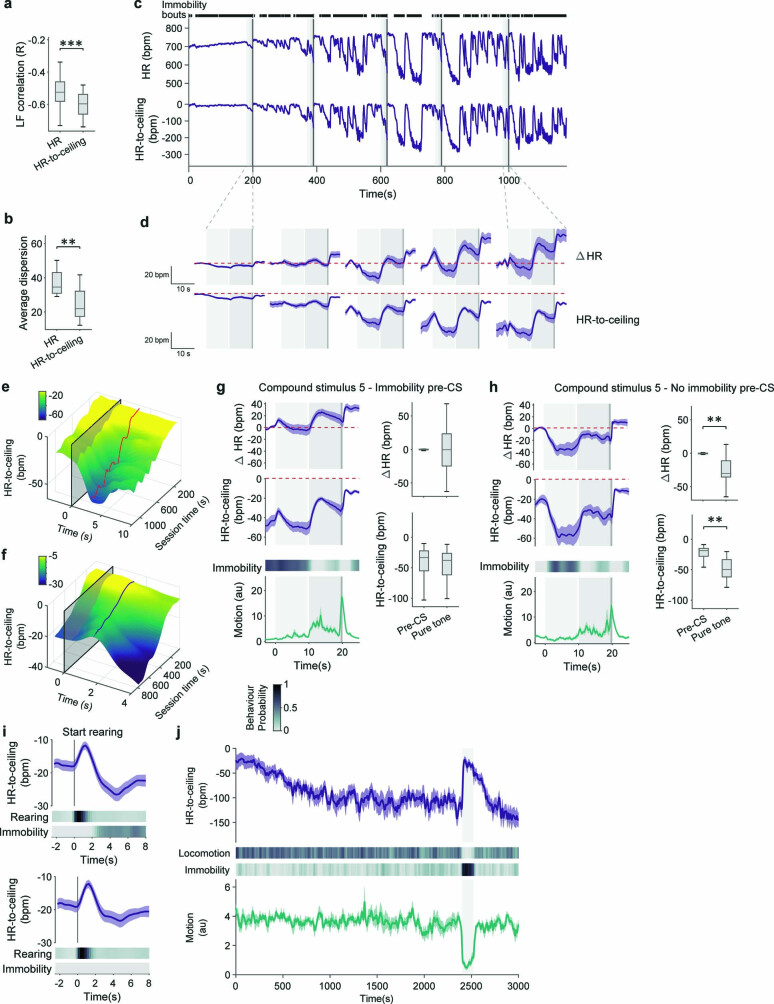
HR-to-ceiling as an alternative to other HR readouts prevents analytical and interpretational confounds, and exposes microstates dependencies. **a**, **b**, HR-to-ceiling is better correlated to LF amplitude (**a**, n = 33 mice, two-tailed Mann–Whitney test) and explains more variability (**b**, n = 33 mice, two-tailed t-test) than raw HR even when ignoring the initial ramping phase. **c**, Example raw HR (top) and corresponding HR-to-ceiling traces (bottom) from a conditioning day 2 recording for a single mouse; black lines on top represent immobility bouts. **d**, PSTHs of ΔHR (top) or HR-to-ceiling (bottom) for the successive CS-US pairings show a progressive increase in the HR changes’ amplitude but also that ΔHR is misleading by not exposing the initial bradycardia and showing the rest as an increase from baseline, unlike HR-to-ceiling which shows it unambiguously (n = 33 mice). Red dashed line shows pre-SCS1 values to help visualization, and grayed areas represent pure tone, white noise, and shock. **e**, **f**, 3D representations showing the increase in amplitude of the immobility- and rearing-associated HR changes in function of the recording time as in Fig. 1f,h, respectively, but with HR-to-ceiling instead of ΔHR. HR-to-ceiling also better captures the global relaxation over time as visible in the baseline. **g**, **h**,PSTHs (left) and matching quantifications (right) for the last CS-US pairings from conditioning day 2 (n = 33 mice), split into trials for which there was immobility before CS onset (**g**) or not (**h**). Because pre-CS immobility is accompanied by low HR, ΔHR suggests opposite HR changes over the CS-US pairing period (**g** and **h**, top curve) depending on whether there was immobility before or not (**g** and **h**, heat map and bottom curve for motion). HR-to-ceiling (**g** and **h**, middle curve) on the other hand allows to clearly interpret the difference as coming for the pre-CS state of the animal while the CS-US pairing leads to similar changes in both cases (immobility or not before). **i**, PSTHs comparing HR-to-ceiling changes accompanying rearing, whether the bout was closely followed by some immobility (top panel) or not (bottom panel), and showing that immobility accounts for some of the post-rearing bradycardia that can be observed on the overall average. **j**, Average curves ±s.e.m. for the CS (pure tone in this case) retrieval after a long baseline in the homecage (n = 8 mice). The CS (grayed area) was presented after the HR-to-ceiling had stabilized at a low value (top curve). All PSTHs shown present mean values ±s.e.m. In box whisker plots, boxes span the first to third quartiles (Q1 to Q3), horizontal line denotes the median, and whiskers show most extreme data points. See Supplementary table 1 for detailed statistical information. **P < 0.01, ***P < 0.001 .

There is a crucial corollary to our finding that the rigidity macrostate affects, for example, cue-induced defensive microstates: quantifying changes that are part of a microstate (for example, bradycardia during immobility) without taking macrostates (for instance, time within the recording) into account leads to confounded results and added variability. To circumvent this issue of added complexity by the interaction of microstates and macrostates, other studies often include handling procedures^26^ or ‘acclimation’ periods before recordings, in an attempt to remove any influence of macrostates experimentally. However, in parallel to evoking repeated and poorly controlled stressful events, this means essentially manipulating the defensive state itself, and ultimately ignoring potentially important aspects of defensiveness. Nevertheless, the slow increase in ‘baseline’ HR level (ceiling) could confound quantifications based on the absolute values. The commonly used moment-to-moment HR differences or changes from a baseline (ΔHR) remove the influence of macrostates analytically at the cost of losing information about absolute HR. We, therefore, introduce a detrended ‘HR-to-ceiling’ measure (Δ between HR and ceiling values, at each time point) as a representation of HR free from the influence of the ceiling macrostate and inter-mice differences in basal HR but allowing for direct comparisons between conditions and/or individuals (Fig. 2a). Strikingly, amplitude of LF oscillations, which reflect complex interactions between the parasympathetic and sympathetic systems^27–29^, correlated more strongly with HR-to-ceiling values than absolute HR (Fig. 2b and Extended Data Fig. 2a). The fact that LF oscillations correlate better with HR-to-ceiling than with absolute HR, therefore, suggests that the HR-to-ceiling measure has not only analytical but also biological relevance. HR-to-ceiling values revealed that bradycardia is present with an extremely low amplitude also during immobility bouts early in the session (SCS1) and also occurs concomitantly with immobility during late tone exposures (SCS5), thereby confirming the global progressive increase in amplitude (Fig. 2c and Extended Data Fig. 2c–e).

Additional analyses revealed that the marked differences between the two quantifications were due to a strong interdependency of microstates. When mice were immobile right before tone exposure, no further HR decrease was observed (Extended Data Fig. 2g,h), which supports the idea that evoked and spontaneous immobility/bradycardia microstates are aspects of a single entity, highlighted here by the continuum between the two. By contrast, locomotion bouts were associated with HR increases only when they were following an immobility bout (Fig. 2d), whereas there was always an increase in HR at the end of an immobility bout, regardless of whether it was terminated by locomotion or nonlocomotor behavior (Fig. 2e). Similarly, the HR decrease following rearing could be attributed mainly to immobility bouts (Extended Data Fig. 2i). This shows that HR increases and decreases do not merely reflect beginning and end of locomotion, respectively, and also that entering or exiting the immobility/bradycardia microstate constitutes the decisive defensive state ‘switch’.

Notably, our data also suggest that an HR decrease (or increase) per se is not specific for a certain microstate, but that the absolute distance to maximal HR at any given time is a major determinant of a microstate, best captured by HR-to-ceiling measure. To further probe the interdependency of microstates and macrostates, we conditioned another cohort of mice and presented them with the CS (pure tone only) in their HC, after a long baseline period. As expected, the long baseline allowed HR to return to low values and, remarkably, the CS presentation led to immobility as a behavioral response accompanied by a strong increase of HR (Fig. 2f and Extended Data Fig. 2j), paralleling earlier results using similar conditions^21,30^.

These results show that cardiac responses, even for similar stimuli and during identical behaviors, depend heavily on the pre-existing state of the animal. Conversely, this demonstrates that cardiac changes differentiate between defensive reactions seemingly identical on the behavioral level.

## Rigidity macrostate effects on microstate dynamics

**Fig. 3:**
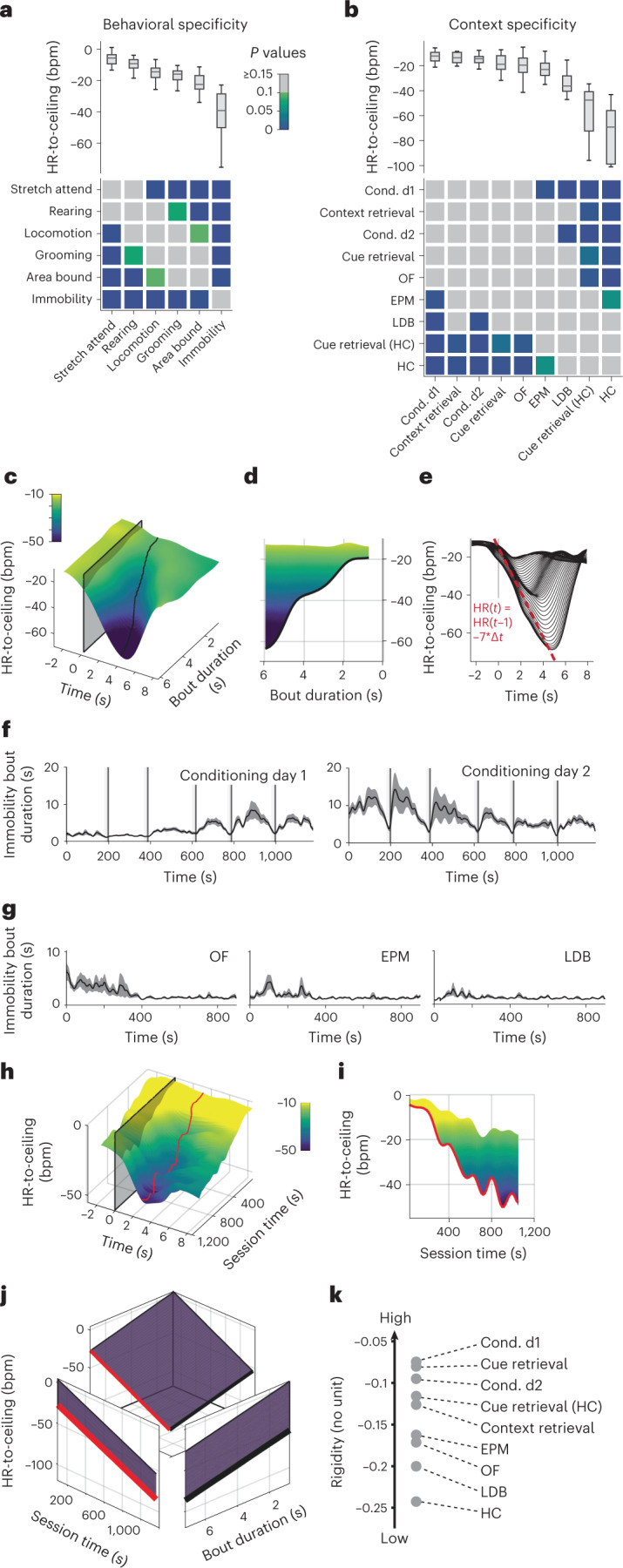
Individual defensive states depend on the context and duration of behavior. **a**, Average values of HR-to-ceiling for different behaviors during conditioning day 2 recordings (top), and grid showing the results of statistical evaluation of the differences between them (bottom; *n* = 33 mice; Kruskal–Wallis followed by post hoc pairwise comparison with Bonferroni correction). **b**, Comparison of the average values of HR-to-ceiling during locomotion in the different paradigms (top) and corresponding statistical analysis (bottom; OF: *n* = 23 mice; EPM: *n* = 20 mice; conditioning day 1: *n* = 30 mice; conditioning day 2: *n* = 33 mice; LDB: *n* = 12 mice; context retrieval: *n* = 5 mice; cue retrieval: *n* = 10 mice; cue retrieval (HC): *n* = 9 mice, HC: *n* = 10 mice; one-way ANOVA followed by post hoc pairwise comparison with Bonferroni correction). **c**, Three-dimensional representation of the immobility-associated decrease in HR-to-ceiling during conditioning day 2 in function of bout duration (*n* = 33 mice), showing that longer episodes are associated with greater bradycardia. **d**, Side view from **c** showing the correlation between immobility bout duration and bradycardia amplitude. **e**, Two-dimensional visualization of **c** showing the conserved kinetics across all bout durations (each line represents a range of bout durations values). **f**,**g**, Mean immobility bout duration ± s.e.m. in function of time, showing stereotypical changes for the different contexts (**f**, OF: *n* = 26 mice; EPM: *n* = 22 mice; LDB: *n* = 12 mice; **g**, conditioning day 1: *n* = 40 mice; conditioning day 2: *n* = 40 mice). **h**,**i**, Three-dimensional representation of the immobility-associated HR decrease during conditioning day 2 in function of the session time (**h**) and 2D side view (**i**), highlighting the dependency on the time within the session. **j**, Fit integrating the amplitude of immobility-associated bradycardia in function of bout duration and time within the session (*R*² = 0.991). The curve in black is the equivalent of **d**, after accounting for the time within the session, while the curve in red is the equivalent of **i** after taking into account the influence of bout duration and its changing values in different contexts. **k**, Values of rigidity estimated at *t* = 10 min from a similar analysis for the different paradigms. The coefficients ranking converges with the sorting of the contexts in terms of immobility- or locomotion-associated HR-to-ceiling values. In box whisker plots, boxes span the first to third quartiles (Q1 to Q3), horizontal line denotes the median and whiskers show the most extreme data points. See Supplementary Table 1 for detailed statistical information.

**Extended Data Fig. 3:**
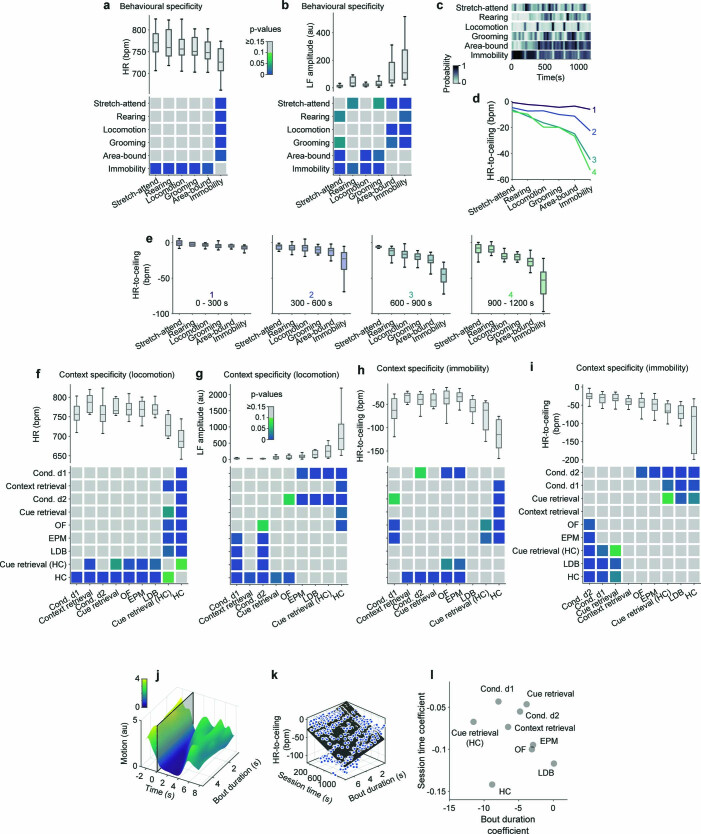
Microstate hierarchies revealed by HR-to-ceiling are recapitulated by LF amplitude but not raw HR, and depend on intrinsic properties of microstates. **a**, Average values of HR for different behaviors during conditioning day 2 recordings (top), and grid showing the results of statistical evaluation of the differences between them (bottom; one-way ANOVA followed by post-hoc pairwise comparison with Bonferroni correction), hinting towards a hierarchy but barely reaching any statistical significance because of high variability (n = 33 mice). **b**, Average values of LF amplitude for different behaviors during conditioning day 2 recordings (top), and grid showing the results of statistical evaluation of the differences between them (bottom; Kruskal-Wallis followed by post-hoc pairwise comparison with Bonferroni correction). The hierarchy is similar to that of HR-to-ceiling values (n = 33 mice). **c**, Probabilities of the different behaviors over the course of conditioning day 2 (n = 33 mice). There is no clear evolution over time that could interact with the progressive rigidity decrease to explain the ranking of themic average HR-to-ceiling values (Fig. 3a). **d**, Average values of HR-to-ceiling for the different behaviors. Each curve (1 to 4) represents a different time range within the conditioning day 2 session (respectively 0–300 s, 300-600 s, 600-900 s and 900–1200 s). While the values are initially (1, top) constrained to a narrow range by the high rigidity, the differences get more pronounced over time, but the ranking of the different behaviors exhibits stability, demonstrating again that the average in Fig. 3a reflects a global difference for the whole conditioning day 2 (RM ANOVA on n = 33 mice: effect of time and behavior, P < 0.001; interaction, P < 0.001) **e**, Similar analyses as Fig.3a, but for the individual time ranges used in (**d**), to better illustrate the ranking of HR-to-ceiling values for the different behaviors is maintained throughout the session. **f**, Comparison of the average values of HR during locomotion in the different paradigms (top), and corresponding statistical analysis (bottom; Kruskal-Wallis followed by post-hoc pairwise comparison with Bonferroni correction). There is a lot of variability and no clear difference nor hierarchy. **g**, Comparison of the average values of LF amplitude during locomotion in the different paradigms (top), and corresponding statistical analysis (bottom; Kruskal-Wallis followed by post-hoc pairwise comparison with Bonferroni correction). The hierarchy parallels that of HR-to-ceiling. **h**, Grand average for HR-to-ceiling values associated with immobility yields paradoxical picture when immobility duration and relative time in the recording are not taken into account (Kruskal-Wallis followed by post-hoc pairwise comparison with Bonferroni correction). **i**, After accounting for bout duration and relative time during the recordings, HR-to-ceiling values associated with immobility also allow to differentiate the contexts (one-way ANOVA followed by post-hoc pairwise comparison with Bonferroni correction). **j**, 3D representation of motion values in function of immobility bout duration, showing the expected correlation (n = 33 mice). **k**, Same fit as in Fig. 3j, with individual average values displayed as dots. **l**, Session time coefficient in function of bout duration coefficient for each paradigm. Apart from HC-related coefficients which seem to not follow the same logic, bout duration coefficients for the other paradigms follow an opposite ranking as for the session coefficients: higher threat contexts are associated with a larger bout duration coefficient. For **f-I & l**, OF: n = 23 mice; EPM: n = 20 mice; conditioning day 1: n = 30 mice; conditioning day 2: n = 33 mice; LDB: n = 12 mice; context retrieval: n = 5 mice; cue retrieval: n = 10 mice; cue retrieval (HC): n = 9 mice, HC: n = 10 mice. In box whisker plots, boxes span the first to third quartiles (Q1 to Q3), horizontal line denotes the median, and whiskers show most extreme data points. See Supplementary table 1 for detailed statistical information.

Consequently, we investigated whether cardiac states differentiate distinct microstates associated with stereotypical defensive behaviors in general. In fact, various defensive behaviors were related to substantially different levels of HR-to-ceiling values (Fig. 3a). Risk assessment behaviors such as stretch-attend posture and rearing were associated with the highest HR-to-ceiling values. Intermediate levels were found for locomotor behaviors, confirming that increases in HR are not simply adjustments to physical needs^31^ (Fig. 3a). Raw HR allowed differentiation only between immobility and other behaviors, whereas LF amplitude values identified more-subtle differences, albeit with less precision than HR-to-ceiling (Extended Data Fig. 3a,b). Because of the strong influence of rigidity changes over time on the HR responses and the intricate relation between behaviors and HR, we checked whether one behavior was predominantly expressed during, for example, the initial period of high rigidity, whereas another behavior was predominantly expressed at the end of the recording during low rigidity. However, temporal distribution of behaviors did not explain the differences observed (Extended Data Fig. 3c), and analyses of behavior over time, while showing the effect of within-session rigidity development, confirmed the differences in average HR-to-ceiling values for the different behaviors (Extended Data Fig. 3d,e). As rigidity decreased, allowing for a wider range of HR values, differences between behaviorally defined microstates increased.

Next, we asked whether microstates reflect the behavioral context in which they occur. We exposed mice to different threat contexts and focused on HR-to-ceiling values associated with locomotion, a behavior present in all context conditions (Fig. 3b). Strikingly, ranked HR-to-ceiling values reflected expected contextual threat levels, with highest values under conditions of concrete threat (conditioned flight paradigm), intermediate values in paradigms with diffuse threat (OF, elevated plus maze (EPM), light–dark box (LDB)) and lowest values in the HC (Fig. 3b). Again, raw HR yielded no differences, whereas LF amplitude measures recapitulated the HR-to-ceiling mean values, albeit with much less variability for shock-related contexts (Extended Data Fig. 3f,g).

By contrast, ranking of contextual influence on immobility was less clear (Extended Data Fig. 3h). We reasoned that this was due to immobility-bout kinetics and found a strong correlation between the bradycardia amplitude and immobility bout duration during conditioning (Fig. 3c,d), with markedly stereotypical kinetics, in particular for the curve’s decreasing segment (Fig. 3e). In addition, immobility-bout duration differentially developed over time in different contexts: it progressively increased during initial threat conditioning day 1 (Fig. 3f, left panel**)**, remained high on the second day of conditioning but showed a decrease in the course of the session (Fig. 3f, right panel). Under diffuse threat conditions (OF, EPM, LDB), immobility-bout durations were slightly longer at the beginning of the recording (Fig. 3g). This suggests that the interaction between both findings (dependency on bout duration and stereotypical bout durations) could influence the results of indiscriminate averaging. In a comparable manner, the progressive changes in rigidity over time (Fig. 3h,i) certainly influence such quantification. Thus, taking into account the time- and context-dependency of immobility-bout duration, we restricted them to certain ranges (last intertrial interval, ≤2.5 s bout duration) to mitigate their influence. The result allowed ranking of the different contexts that resembled the context distribution for locomotion, but with comparatively reduced variability (Extended Data Fig. 3i).

This prompted us to find a way of integrating within the same analysis the two major influences—bout duration and time within the recording—on immobility-associated bradycardia. We extracted the peak bradycardic values (that is, the lowest HR-to-ceiling value during a given immobility bout) for immobility episodes spanning the possible ranges of bout durations and time within the recording and fit a simple model to them (Fig. 3j and Extended Data Fig. 3k). The result was a linear equation with one coefficient (*A*_s_), accounting for within-session changes in HR amplitudes, thereby accounting for the slow rigidity macrostate, and another for bout duration (*B*_d_). It followed that ${\mathrm{Bradycardia}}_{\mathrm{{IB}}} = A_{\mathrm{s}} \times {{{\mathrm{t}}}} + B_{\mathrm{d}} \times {\mathrm{Duration}}_{\mathrm{{IB}}}$, with Bradycardia_IB_ standing for peak amplitude of HR deceleration during a given immobility bout and Duration_IB_ representing the length of the immobility bout, and *t* the time at which it occurred (Extended Data Fig. 3l; detailed explanations in the Methods section). This approach allowed us to differentiate the contribution of bout duration and session time on the immobility-bradycardia microstate. By comparing the coefficients between the different paradigms, we were able to find a similar ranking as for the averages (for example, Fig. 3b) for the session coefficients, which can be seen as a relative rigidity measurement (that is, smaller coefficients mean that the time within the session is associated with moderate increase in bradycardia amplitude, and conversely). As such, paradigms with expected high-threat levels (for example, conditioned flight) had the lowest values, and therefore the highest rigidity overall (Fig. 3k).

We were, therefore, able to confirm that specific behaviors are associated with particular cardiac profiles, but also that the immobility-related microstate commonly termed ‘freezing’ is not a homogenous and static event determined only by its probability of occurrence. It crucially exhibits intrinsic behavioral properties, that is, individual bout duration associated with dynamic cardiac changes. This is supported by the fact that immobility-bout duration was not random, but instead exhibited context- and time-dependent patterns. Moreover, we show that expression of microstates is heavily influenced by slower underlying macrostate changes, which partially depend on the context in which they occur. Overall, this emphasizes their value in characterizing integrated defensive states.

## Integrated defensive states encode contextual threat levels

**Fig. 4:**
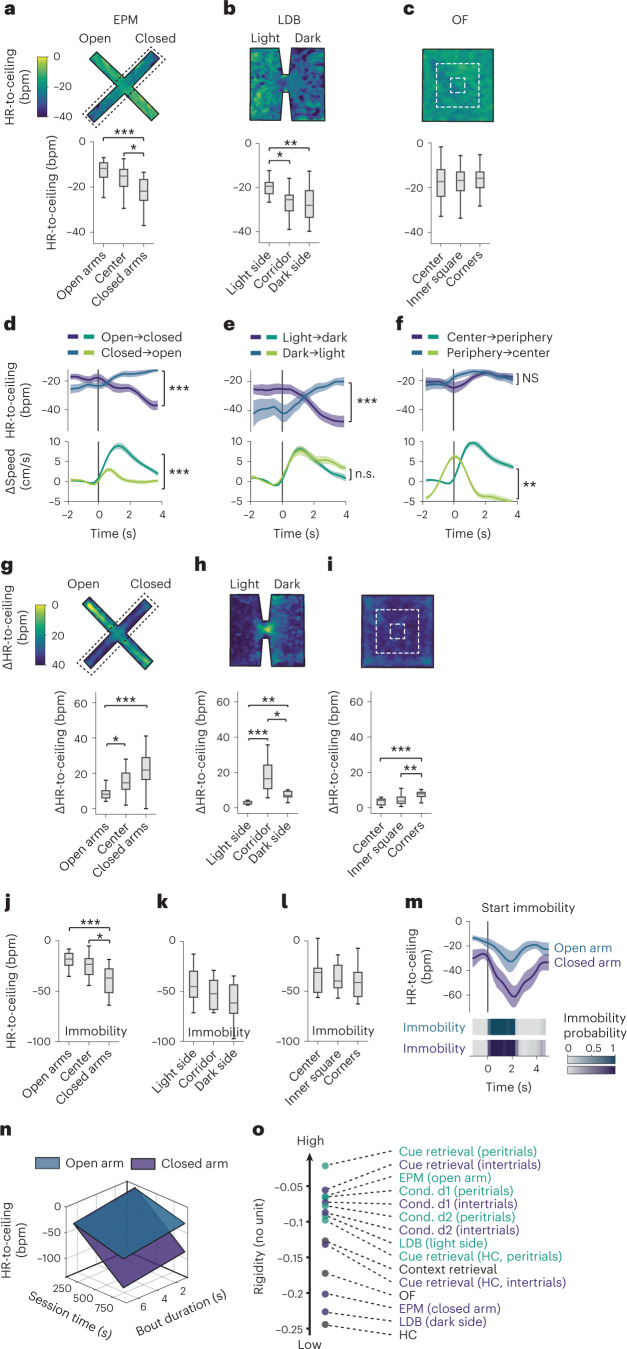
Integrated defensive states encode contextual threat levels. **a**–**c**, Heat map representations of the mean HR-to-ceiling values in the different contexts (top) and corresponding quantification for discrete subareas (bottom; **a**, EPM: *n* = 20 mice, one-way ANOVA followed by post hoc pairwise comparison with Bonferroni correction; **b**, LDB: *n* = 12 mice, one-way ANOVA followed by post hoc pairwise comparison with Bonferroni correction; **c**, OF: *n* = 23 mice, one-way ANOVA). **d**–**f**, PSTHs for transitions between low-threat and higher-threat subareas, showing that the corresponding average locomotion bouts (bottom curves, Δspeed ± s.e.m.) are associated with direction-specific HR-to-ceiling changes (top curves, HR-to-ceiling ± s.e.m.) in the EPM (**d**; *n* = 20 mice, two-tailed *t*-test) and LDB (**e**; *n* = 12 mice, two-tailed *t*-test), but not the OF (**f**; *n* = 23 mice, two-tailed Mann–Whitney test). *t*_0_ corresponds to the start of the area transition locomotion bouts. **g**–**i**, For each panel, heat map showing the average Δ between highest and lowest HR-to-ceiling values expressed within a recording in each position of the whole context (top), and the corresponding quantifications (bottom; EPM: *n* = 20 mice (**g**), LDB: *n* = 12 mice (**h**), OF: *n* = 23 mice (**i**); all Kruskal–Wallis followed by post hoc pairwise comparison with Bonferroni correction). **j**–**l**, Subarea-specific values of HR-to-ceiling during immobility (**j**, EPM: *n* = 20 mice, one-way ANOVA followed by post hoc pairwise comparison with Bonferroni correction; **k**, LDB: *n* = 12 mice, one-way ANOVA; **l**, OF: *n* = 23 mice, one-way ANOVA). **m**, PSTH illustrating that for immobility bouts of matching durations (bottom), the amplitude of immobility-associated bradycardia (mean ± s.e.m., top) is lower in the open than in the closed arms of the EPM. **n**, Sheet analysis of the immobility-associated bradycardia for closed versus open arms (respectively, *R*² = 0.989 and *R*² = 0.97), confirming a globally smaller amplitude in the open arms (*n* = 20 mice, one-tailed *F* test, *P* < 0.001,). **o**, Values of rigidity estimated at *t* = 10 min from sheet analyses performed on the different contexts, differentiating between high versus low-threat subareas or periods. In box whisker plots, boxes span the first to third quartiles (Q1 to Q3), horizontal line denotes the median and whiskers show the most extreme data points. See Supplementary Table 1 for detailed statistical information. *P < 0.05, **P < 0.01, ***P < 0.001.

**Extended Data Fig. 4:**
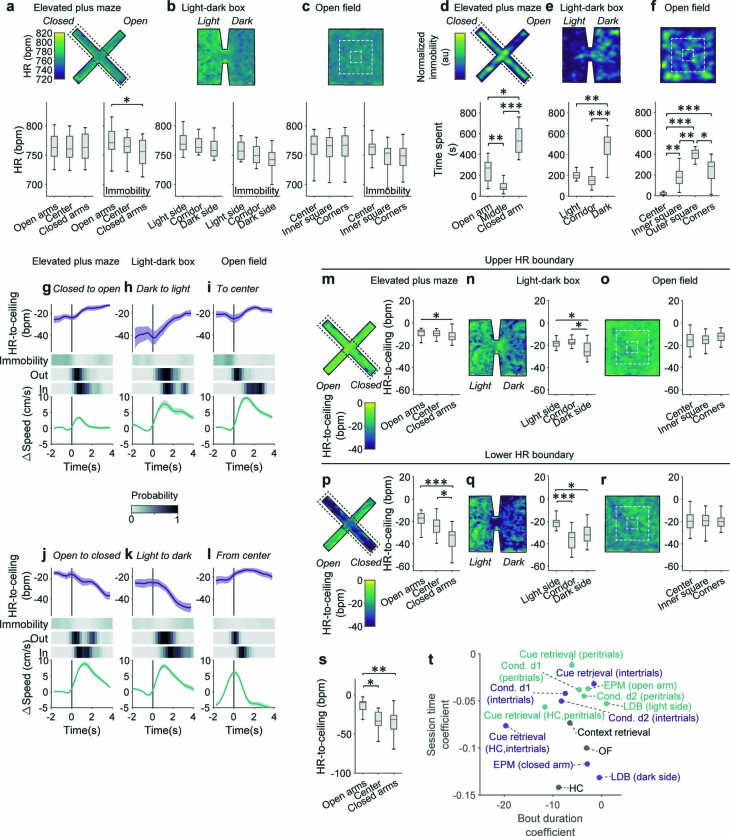
HR-to-ceiling, but not raw HR, reflects subarea-related macrostates, their influence on microstates, and transitions. **a-c**, Heat map representations of the mean HR values in the different contexts (top), corresponding quantification for discrete subareas (bottom, left boxplot; EPM: n = 20 mice, one-way ANOVA; LDB: n = 12 mice, one-way ANOVA; OF: n = 23 mice, one-way ANOVA), and subarea-specific values of HR during immobility (bottom, right boxplot; EPM: n = 20 mice, one-way ANOVA followed by post-hoc pairwise comparison with Bonferroni correction; LDB: n = 12 mice, one-way ANOVA; OF: n = 23 mice, Kruskal-Wallis). **d-f**, Heat map representations of the normalized immobility probability in the different contexts (top), corresponding quantification for discrete subareas (bottom, EPM: n = 20 mice, one-way ANOVA; LDB: n = 12 mice, one-way ANOVA; OF: n = 23 mice, one-way ANOVA). **g-l**, PSTHs showing separately mean HR-to-ceiling ±s.e.m. (top) and speed changes ±s.e.m. (bottom) for the transitions from low threat areas to higher threat areas (top row) or the opposite (bottom row), for EPM (n = 20 mice), LDB (n = 12 mice) and OF (n = 23 mice). The heat plots below immobility probability show the cumulative distribution for all the averaged transitions of the exit from the start area (‘Out’) and the arrival in the target area (‘In’). Note that both modest immobility probability, and conversely, the ongoing locomotion, are similar for both directions, or that higher speed is associated with the bradycardic response, and that locomotion is therefore unlikely to account for the changes in HR-to-ceiling observed. **m-r**, Heat map representations of the maximum (top row) as well as minimum (bottom row) HR-to-ceiling values in the different contexts, and corresponding quantification for discrete subareas (EPM: n = 20 mice, Kruskal-Wallis (top) and one-way ANOVA (bottom) followed by post-hoc pairwise comparison with Bonferroni correction; LDB: n = 12 mice, Kruskal-Wallis followed by post-hoc pairwise comparison with Bonferroni correction; OF: n = 23 mice, one-way ANOVA). **s**, HR-to-ceiling average values associated with immobility episodes in subareas of the EPM (n = 20 mice, Kruskal-Wallis followed by post-hoc pairwise comparison with Bonferroni correction); similar to Fig. 4j, except that only immobility episodes of similar duration were selected here, to confirm that the effect observed is not due to a potential difference in bout duration between subareas. **t**, Session time coefficient in function of bout duration coefficient for each paradigm and/or subperiod/subarea. While the ranking for session coefficient follows extremely well the intuitive threat levels, bout duration coefficient is likely to be influenced by other factors that are not readily visible in this analysis. In box whisker plots, boxes span the first to third quartiles (Q1 to Q3), horizontal line denotes the median, and whiskers show most extreme data points. See Supplementary table 1 for detailed statistical information. *P < 0.05, **P < 0.01, ***P < 0.001.

Our data demonstrated that HR exhibits high sensitivity to different threat levels induced by cues and contexts. Standard paradigms for fear and anxiety, solely developed based on behavioral readouts, use specific context designs to infer spatially-dependent threat levels^32^. We, therefore, asked next whether detrended HR-to-ceiling values would also report contextual subregions in classical tests evoking diffuse threats. Strikingly, we found a gradient of HR-to-ceiling values from low to high-threat areas in EPM and LDB assays (Fig. 4a,b). Equivalent analyses based on the raw HR did not yield any significant differences (Extended Data Fig. 4a,b). Surprisingly, no differences were observed between the center and periphery of the OF test (Fig. 4c and Extended Data Fig. 4c), even though thigmotaxis and immobility were in line with standard expectations (Extended Data Fig. 4d–f). This unexpected result suggested that cardiac activity could reflect relative homogeneity (for example OF) or explicit compartmentalization (for example, EPM and LDB) of threat contexts and prompted us to ask whether HR differences between certain subregions predicted the existence of state switches during transitions from one to the other context compartment. On the behavioral level, no such switch was visible, since mice exhibited locomotion patterns that differed in magnitude but not in direction when transitioning from low- to high-threat compartments and vice versa (Fig. 4d–f, lower panels, and Extended Data Fig. 4g–l, lower panels). By contrast, transitions from high- to low-threat areas were accompanied by marked decreases in HR, whereas transitions from low- to high-threat areas were concomitant with increases in HR, reflecting state switches mediated by the different subareas (Fig. 4d–f, upper panels, and Extended Data Fig. 4g–l, upper panels).

What is the nature of these context-dependent states? We hypothesized that contextual threat levels evoked a macrostate mechanistically similar to rigidity, that is imposing a restriction of HR-to-ceiling values in high-threat areas. This became apparent when instead of taking mean values per bin, deltas between maximum and minimum of the detrended HR-to-ceiling (Fig. 4g–i and Extended Data Fig. 4m–r) were used to identify subarea-specific changes. Decisively, smallest HR ranges are apparent in high-threat areas, such as open arms in the EPM, light side in the LDB and center in the OF (Fig. 4g–i). These findings were reminiscent of the contracted HR variability, that is, the high rigidity macrostate at the beginning of a threat conditioning session (Fig. 1d–g). We, therefore, looked at the amplitude of immobility-associated bradycardia across the different subareas, which was substantially smaller in the high-threat areas (EPM open arms, LDB light side) than in low-threat areas (EPM closed arms, LDB dark side; Fig. 4j–l) mimicking changes observed during fear conditioning. Crucially, this did not rely on different intrinsic behavioral properties, as it stood true even when we matched immobility bout duration (Fig. 4m and Extended Data Fig. 4s). Applying the integrated analysis of immobility-associated bradycardia confirmed this finding, as open and closed arms presented very distinct profiles, with higher rigidity observed in the open arm (Fig. 4n and Extended Data Fig. 4t). We hypothesized that this could be a general property of HR that would reflect threat level and performed a similar analysis on the different paradigms after differentiating low- versus high-threat periods (that is, inter-CS intervals versus peri-CS intervals), or low- versus high-threat areas as for the EPM. The updated ranking met those expectations, demonstrating that rigidity globally reflects threat level that in turn affects its development over time (Fig. 4o and Extended Data Fig. 4t). Together, the integrated defensive state analysis reflected subarea-specific differences of threat level in classical fear and anxiety tests.

## *Chx10*^+^ neurons in the PAG function as a microstate generator

If such ubiquitous interactions between microstates and macrostates define defensive states on the level of the associated responses, we reasoned that they could help to pinpoint neuronal circuit elements mediating defensive states, such that the evoked responses should present similar properties as in the naturally occurring reactions. We, therefore, used optogenetic manipulations of specific neural circuit elements involved in mediating defensive behaviors. Previous studies assigned such a role to glutamatergic neurons in subregions of the midbrain PAG—a key region for the defense reaction—and supported a labeled line-like mechanism of functionally defined PAG output pathways^33,34^. Glutamatergic neurons of the lateral and ventrolateral PAG, characterized by the expression of vesicular glutamate transporter 2 (*Vglut2*), mediated defensive behaviors ranging from flight to threat-induced immobility, that is, freezing, the latter associated specifically with glutamatergic ventrolateral PAG neurons projecting to the medullary magnocellular nucleus and expressing *Chx10* (refs. ^33–35^). Notably, these studies left unanswered the question of whether and how these circuit elements take part in mediating the integrated defensive response, a role that has long been postulated for PAG circuits^6–8,36,37^.

**Fig. 5:**
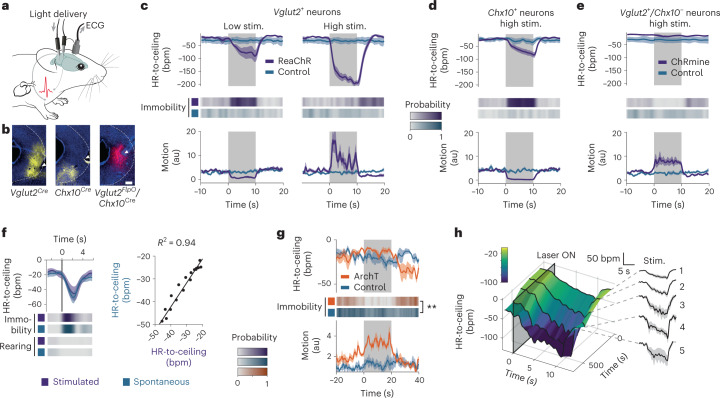
Specific circuit elements in the midbrain PAG mediate integrated defensive microstates. **a**, ECGs are recorded in freely moving mice while light stimulations are delivered to optogenetically activate specific vlPAG neuronal populations. **b**, Representative microscope images showing opsin expression and optic fiber placement for vlPAG optogenetics experiments (scale bar = 200 µm). **c**, Optogenetic stimulation of *Vglut2*^+^ neurons in the vlPAG led to different microstates depending on the stimulation intensity. Both caused a pronounced decrease in HR (top curves), but while the low-intensity stimulation produced immobility (probability plot, middle), also visible via a decrease in motion (bottom curve), the high-intensity stimulation led to the expression of active behaviors instead (both *n* = 8 versus controls, *n* = 5; two-tailed *t*-test for low intensity, two-tailed Mann–Whitney *U* test for high intensity). **d**, Light activation of vlPAG *Chx10*^+^ neurons evoked a defensive reaction resembling the one evoked by low-intensity stimulation of glutamatergic neurons (**c**) and spontaneous episodes, characterized by bradycardia and immobility (*n* = 8 versus controls, *n* = 5). **e**, ChRmine-mediated activation of the *Vglut2*^+^/*Chx10*^*−*^ neurons in the vlPAG recapitulated the behavioral activation seen during high-intensity stimulation of the global *Vglut2*^+^ population, but without any bradycardia. **f**, Bradycardic responses evoked by optogenetic stimulation of *Chx10*^+^ cells (*n* = 8 mice) present the same dynamic and amplitude as spontaneous immobility episodes (left curves) with similar immobility bout duration (heat maps, middle), as shown by the correlation (right, correlation of the time-matched HR-to-ceiling values during immobility between spontaneous and evoked episodes). **g**, Optogenetic inhibition of the *Chx10*^+^ neurons interrupts context retrieval-driven immobility, suggesting again their involvement in the naturally occurring defensive immobility bouts (*n* = 5 versus controls, *n* = 4; RM ANOVA; see Supplementary Table 1 for details). **h**, Three-dimensional representation of the amplitude of HR decrease evoked by optogenetic stimulation of the *Chx10*^+^ neurons (*n* = 8 mice) in function of the relative time from the beginning of the recording (left), showing a progressive increase, also visible on the single PSTH curves (right), and suggesting it is sensitive to the rigidity macrostate in a similar manner as spontaneous immobility episodes. All PSTHs shown present mean values ± s.e.m. Mouse image in **a** adapted from SciDraw. **P < 0.01.

**Extended Data Fig. 5:**
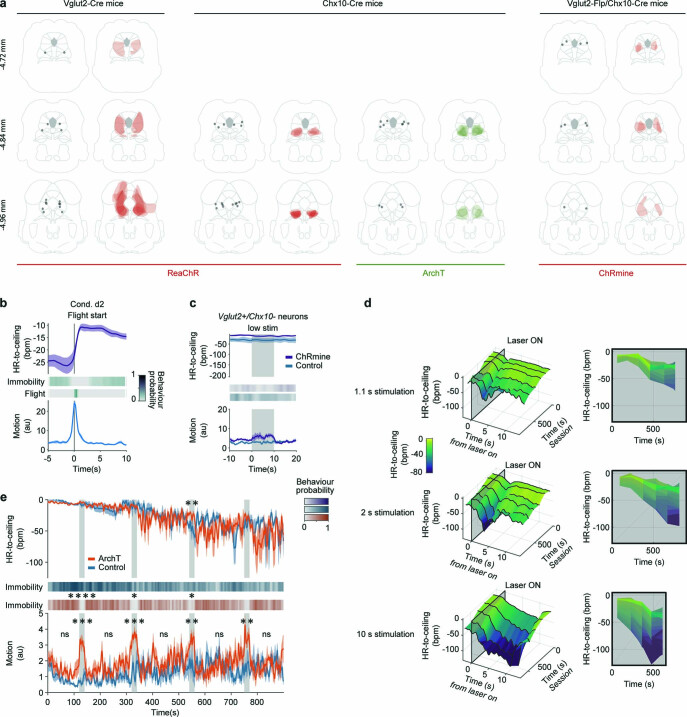
Histology for the optogenetics experiment and rigidity effect generalization to different stimulation protocols. **a**, For each combination of mouse line and viral approach used for the optogenetics experiments, one line per brain level, where the left midbrain section shows the fiber placement and the right one depicts viral expression as overlay for all the mice. **b**, PSTHs showing the average HR (top) and behavioral responses (middle and bottom) associated with flight during conditioning day 2 (n = 33 mice). **c**, PSTHs depicting the responses evoked by a low intensity stimulation of ChRmine-expressing *Vglut2*+*/Chx10-* neurons in the vlPAG. Only a mild behavioral activation was visible on the average motion curve (bottom). **d**, 3D representations of the amplitude of HR decrease evoked by optogenetic stimulation of the *Chx10*+ neurons (n = 8 mice) in function of the relative time from the beginning of the recording (left panels), and corresponding 2D views (right panels), for the 1.1, 2 and 10 s stimulations. The amplitude of bradycardia increases with time, revealing it is affected by rigidity even for more modest stimulation durations, in a linear manner. **e**, Average traces for the whole recordings for the optogenetic inhibition of the *Chx10*+ neurons from the vlPAG during context retrieval, showing the inhibition of immobility by the stimulations (gray areas; n = 5 for ArchT vs n = 4 for controls, RM ANOVAs, see Supplementary table 1 for details). The effects on immobility and motion are more pronounced at the beginning of the recording because immobility probability naturally decreases over time during the context retrieval session. There were no significant differences in baseline or between-trials motion. HR differences are however not very pronounced at early time points because of the usual high *rigidity* at the beginning of the recordings. PSTHs and average traces present mean values ±s.e.m. *P < 0.05, **P < 0.01, ***P < 0.001.

We equipped naive *Vglut2*^*Cre*^, *Chx10*^*Cre*^ and *Vglut2*^*FlpO*^*/Chx10*^*Cre*^ mice with ECG electrodes (Fig. 5a), and locally injected an adeno-associated viral vector (AAV) into the vlPAG to Cre-dependently express ReaChR (Fig. 5b and Extended Data Fig. 5a), a red-light activatable, excitatory optical actuator^38^. Stimulating *Vglut2*^+^ neurons evoked intensity-dependent responses: low intensities led to immobility and bradycardia, whereas higher intensities led to mixed flight and transient immobility and bradycardic HR responses (Fig. 5c and Supplementary Video 2). Optical activation of the *Chx10*^+^ population resulted in robust immobility and bradycardia (Fig. 5d and Supplementary Video 3). This strongly suggested that *Chx10*^+^ neurons are a subgroup of glutamatergic vlPAG neurons mediating a particular defensive microstate, that is, immobility concomitant with bradycardia. Confirming this distinct role, specific optical activation of *Vglut2*^+^*/Chx10*^*−*^ via a double-conditional, intersectional approach recapitulated the behavioral activation seen when activating the entire *Vglut2*^+^ neuronal population but failed to produce any cardiac effect (Fig. 5e and Supplementary Video 5). This was in contrast to HR decreases evoked by optoactivation of the entire *Vglut2*^+^ neuron population or the tachycardic response associated with spontaneous flight (Extended Data Fig. 5b). Whereas activation of all glutamatergic vlPAG neurons with high intensity resulted in a rather non-natural state of strong locomotor behavior concomitant with HR decrease, the *Chx10*-mediated effect resembled the immobility-bradycardia microstate we had characterized before (Fig. 1f). Using different optogenetic protocols first enabled us to confirm that the amplitude of the evoked-bradycardia did indeed increase with stimulation duration, with the same relationship as in spontaneous episodes derived from animals which underwent an OF test without optogenetic perturbation (Fig. 5f and Extended Data Fig. 5d). Inhibition of *Chx10*^+^ neurons during a context threat memory retrieval blocked sustained immobility (Fig. 5g, Extended Data Fig. 5e and Supplementary Video 4). To add further construct validity to these findings, we asked whether the evoked and spontaneous microstates were similarly affected by rigidity. Across repeated optical stimulation of the same length and intensity, the amplitude of the evoked-bradycardia linearly increased in function of time from the first to the fifth trial (Fig. 5h and Extended Data Fig. 5d), demonstrating that like the natural immobility bradycardia microstate, the evoked microstate was sensitive to rigidity. These data obtained by targeted perturbation of PAG circuit elements within our defensive state framework identify *Chx10*^+^ neurons as generators of an integrated defensive microstate, characterized by immobility and bradycardia.

## Multidimensional analysis reveals overarching defense states

**Fig. 6:**
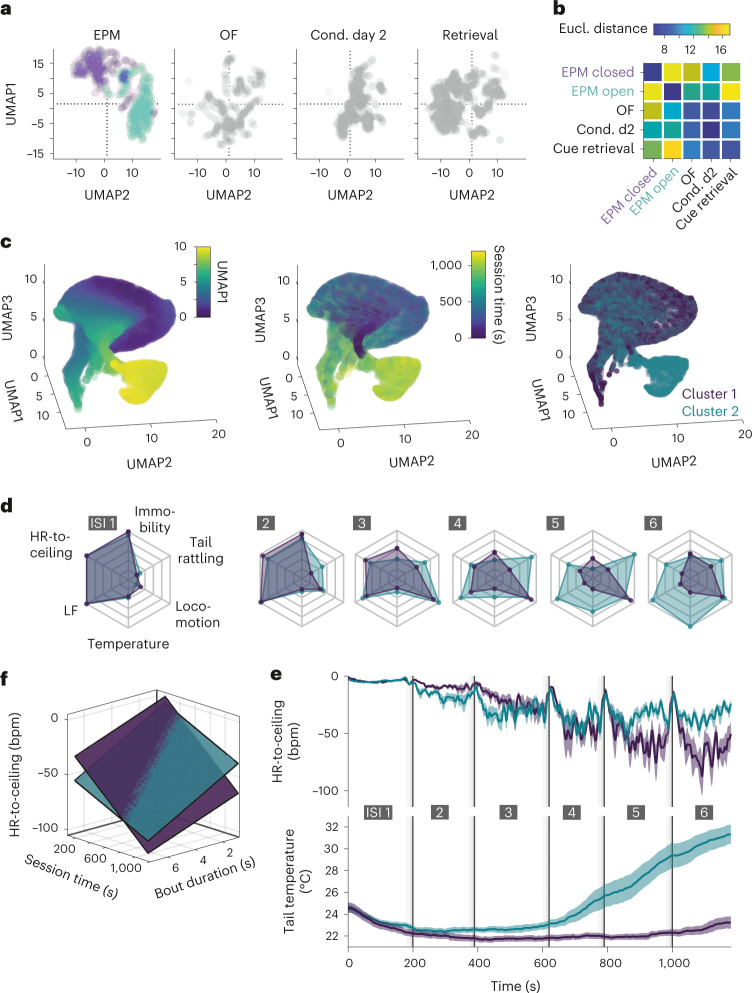
Multidimensional analysis reveals overarching defensive states. **a**, Two-dimensional representation of the UMAP dimensionality reduction of secondary readouts of interest HR-to-ceiling, immobility bout duration, immobility-associated bradycardia, LF amplitude during locomotion) extracted for different paradigms. The two-colored sub-clouds for the EPM correspond to the open-arm and closed-arm data points. **b**, Matrix of the average Euclidean distances between the different contexts corresponding to the UMAP results in **a**, informing about states space relative proximity (average of 250 UMAP runs). **c**, Exploratory 3D plots of UMAP dimensionality reduction applied to a set of raw readouts from conditioning day 2 (including tail temperature) suggested the existence of a subcluster (left, color coding for UMAP dimension 1 for better readability), which seemed to coexist with a main cluster later during the recordings (middle). The results from a BIC-guided k-means clustering of mice averages produced two clusters of mice that corresponded to the previously suspected clusters (right). **d**, Spider charts showing the average values of several readouts over the six ISI for the two groups of mice identified (respectively, *n* = 14 versus *n* = 15), with progressively pronounced autonomic and behavioral differences. **e**, Average curves of mean HR-to-ceiling ± s.e.m. and mean tail temperature ± s.e.m., corresponding to the clusters visible in **c**. The higher average values of HR-to-ceiling from the mid-recording on in the group with an increase in tail temperature suggests a higher rigidity. **f**, Sheet analysis confirms that the cluster of mice with an increase in tail temperature presents an overall higher rigidity, with different dynamics (cluster 1, no temperature increase, *R*² = 0.983, *n* = 14; cluster 2, temperature increase, *R*² = 0.98, *n* = 15; comparison, one-tailed *F* test, *P* < 0.001). This underlines that added readouts can help identify further underlying integrated macrostates.

**Extended Data Fig. 6:**
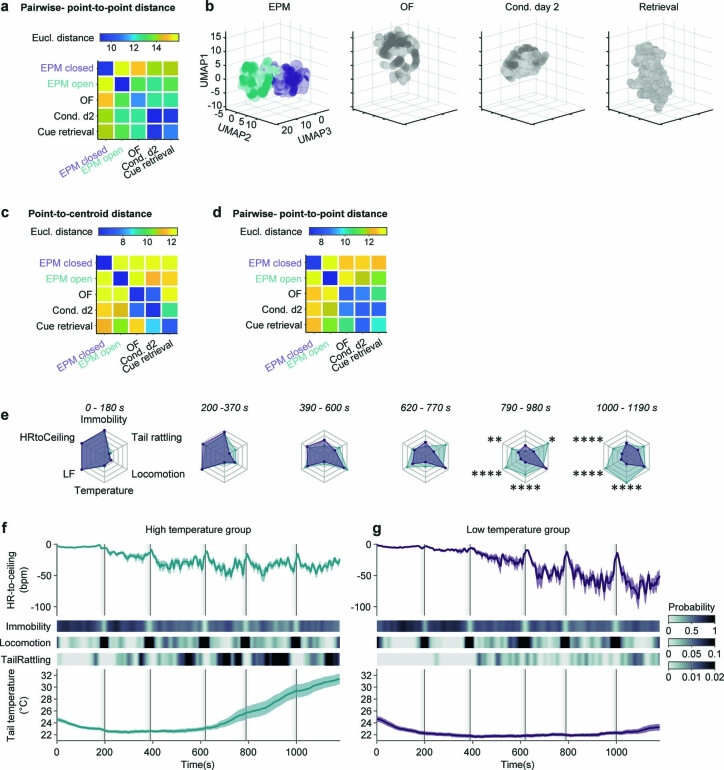
Extension to different metrics and dimensionalities of the ‘state space’-defining methodology and detailed representation of the raw data from the diverging subgroups. **a**, Matrix showing the average pairwise Euclidean distance between single data points for the different contexts corresponding to the UMAP results in Fig. 6a (average of 250 UMAP runs). **b**, 3D representation of the UMAP dimensionality reduction of secondary readouts of interest (ceiling, immobility bout duration, immobility-associated bradycardia, LF amplitude during locomotion) extracted for different paradigms (similar to Fig. 6a, but new analysis with a reduction to three dimensions instead of two). The two-colored sub-clouds for the EPM correspond to the open arm and closed arm data points. **c-d**, Matrix showing the average Euclidean distance between each contexts’ data points and the others’ centroids (**c**) or average pairwise Euclidean distances between single data points (**d**), corresponding to the UMAP results in **b** (averages of 250 UMAP runs). **e**, Spider charts showing the average values of several readouts over the 6 ISI for the two groups of mice identified (respectively n = 14 vs n = 15), with progressively pronounced autonomic and behavioral differences (two-way RM ANOVA followed by Sidak’s multiple comparisons test). **f**, **g**, Average curves of HR-to-ceiling ± (top), immobility, locomotion, and tail rattling probabilities (heat plots in the middle) and tail temperature (bottom) for the two groups of mice, one of which presented an increase in tail temperature (**f**, n = 14) while the other did not (**g**, n = 15). Whole average traces present mean values ±s.e.m. *P < 0.05, **P < 0.01, ****P < 0.0001.

Using behavioral and cardiac autonomic readouts enabled us to define integrated defensive microstates and macrostates. To ensure generalization and translation of this cardio-behavioral framework, we next aimed to integrate multiple parameters derived from behavioral and cardiac readouts commonly used in fear and anxiety research. We selected four variables, reflecting the main states present in our conditions (that is, HR-to-ceiling, immobility bout duration, immobility-associated bradycardia and LF amplitude during locomotion), for which we processed average values in function of time for each paradigm. Subsequent ‘Uniform Manifold Approximation and Projection for Dimension Reduction’ (UMAP^39^) showed that different behavioral paradigms cover different 2D (Fig. 6a) and 3D (Extended Data Fig. 6b) subspaces, a phenomenon also visible between the open and closed arms of the EPM (Fig. 6a,b and Extended Data Fig. 6a–d). These findings demonstrate that combined analysis of multiple readouts allow capture of a general defensive state, associated with a specific context, time or both.

Because defensive states encompass adaptive changes beyond cardio-behavioral dimensions, we lastly tested whether additional state dynamics could be revealed by integration of more autonomic and raw-data readouts, without making use of our understanding of some of the underlying states as before. We performed UMAP analysis of speed, motion, HR-to-ceiling, LF amplitude and thermographically recorded tail temperature obtained during the conditioned flight paradigm. Interestingly, a subcluster was visible on the initial view (Fig. 6c, left), which seemed to develop over time of the behavioral session (Fig. 6c, middle). We used the Bayesian Inference Criterion (BIC) to determine whether averages per mouse could differentiate groups, followed by k-means clustering. This procedure identified two clusters (Fig. 6c, right) corresponding to two groups of mice that diverged particularly regarding their thermoregulatory and cardiac profiles (Fig. 6d,e and Extended Data Fig. 6e–g). Integrated analysis of the immobility-associated bradycardia confirmed major differences in terms of rigidity development over the course of the session, with the group of mice displaying an increase in tail temperature presenting limited decrease of the rigidity over time, thus pointing toward putative macrostate dynamics dependent on inter-individual differences (Fig. 6f). Overall, multidimensional analysis extended our previous findings (Fig. 4) by revealing overarching contextual macrostates associated with different behavioral paradigms and characterized by inter-individual differences in state dynamics.

## Discussion

Our data demonstrate that defensive states consist of integrated and interdependent processes, as previously and intuitively postulated. Based on the state-of-the-art behavioral analysis and the detailed characterization of the technically simple readout HR, we developed a framework that integrates behavioral and cardiac components and reconciles the seemingly contradictory findings in the literature on threat-induced HR changes. We provide strong evidence for the existence of and interaction between short-lasting microstates and macrostates with slower dynamics as important determinants of defensive states.

Our work reconciles the long-standing dispute in the field of whether threat exposure in general, and the immobility state termed freezing in particular, is associated with tachy- or bradycardia. We show that both responses occur and that they crucially depend on context-related internal macrostates such as ceiling and rigidity and the preceding microstate. The initial responses to threat are macrostate changes that promote overall tachycardia. On the background of this long-term change, an important defensive microstate is characterized by immobility and bradycardia. This microstate is induced by contextual and cue-induced threats, but we show that the composition of its behavioral and cardiac components can vary greatly with respect to immobility bout duration and HR-to-ceiling values. This reflects the influence of macrostates on a readout that is commonly used as a single entity to quantify fear, that is ‘freezing’ behavior^40–42^. Thus, our data demonstrate the existence of different defensive states associated with similar readouts such as immobility or HR decrease. When binarizing defensive states into freezing versus nonfreezing periods, or when integrating behavioral data over long periods of time (for example, by using average freezing values), the important correlations between microstates and macrostates are lost, thereby preventing potential attributions of neuronal activity with a particular readout. By taking into account the integrated and dynamic nature of the threat-related immobility—commonly termed as the freezing defensive state—we were able to identify the underlying circuit elements, that is, excitatory *Chx10*^+^ neurons within the vlPAG. This shows how combining classic gain- and loss-of-function approaches with previously identified microstates and macrostates properties can strengthen the interpretation of a certain circuit element being involved in a natural state. Overall, our findings refine the ‘labeled line’ concept^4,9,43,44^, which postulates the existence of functionally defined neuronal pathways, by addressing the multitude of highly dynamic and interacting brain functions in the context of defense against threats.

Our results reveal that for several integrated defensive states, the average distance to an approximated maximal HR (HR-to-ceiling) is the most relevant characteristic. This suggests that physiologically, there is not a ‘fixed’ signal systematically leading to HR change of similar amplitude, but that HR is adjusted to a particular set point for individual microstates that are associated with specific defensive behaviors. Notably, these microstate-related set points are affected by the (sub)context-dependent changes in the rigidity macrostate. Both aspects are captured by our framework, which discretizes HR data to unravel semistable states, and which includes temporal dynamics, which can characterize states of their own. This is most evident in the described ceiling and rigidity macrostates, relating to the upper boundary and range of cardiac output, respectively. Notably, ceiling is predominantly determined by cardiac function and thus may reflect sluggish peripheral endocrine processes, whereas the richer dynamics of rigidity report the interaction between cardiac and behavioral defensive states. The term rigidity originates from our initial hypothesis that although HR can reflect moment-to-moment changes in defensive states, the amplitude does not necessarily reflect only for example a discrete ‘fear level’ at a given time but is also constrained by higher-order processes. This was further confirmed and supported by our data: rigidity is a neuro-autonomic phenomenon that opposes HR changes. An analogy would be a stretched rubber band that resists deflection pressure (Extended Data Fig. 1d). Mechanistically, it most likely reflects changes at the level of autonomic balance, which constrains the changes in HR that can be achieved—very much like the shifts described for the baroreflex curve^45^. In addition, our finding that LF oscillations of HR, which have been associated with emotions in humans^25^, are sensitive to rigidity strongly suggests that these two processes share an underlying mechanism, such as baroreceptor reflex function. *Chx10*^+^ neurons of the vlPAG serve as microstate generators, but they do not affect rigidity and are unlikely to regulate baroreflex function directly. Consequently, although they always send the same signal (same deflection force onto the rubber band) to their downstream regions, their action would be strongly modulated by rigidity (different preload on the rubber band), and thus not always yield the same integrated microstate. Thus, it will be important in future studies to determine how brainstem and higher-order circuits control temporal dynamics of central and peripheral macrostates. Nervous systems have been modeled as near-chaotic systems stabilized by attractors^46^. Our data suggest similar state dynamics, that is main and partial attractors as macrostates and microstates, respectively, for behavioral and cardiac readouts. It is conceivable that this is closely related to fractal characteristics of HR, which exhibit rigidity after pharmacological stimulation^47^. Overall, it reaffirms our framework’s adequacy in providing a window into higher-order brain functions such as defense against threat. Notably, defensive state dynamics involve bi-directional brain–body communication, that is, somato- and visceromotor control and interoception^48^.

From a methodological perspective, we show that when solely relying on behavioral readouts, the complexity of ‘hidden’ state dynamics is lost. Moreover, HR can indicate distinct threat-dependent state shifts even when there are no observable changes in behavior. However, higher-order defensive states derived from human emotions such as ‘fear’ and ‘anxiety’ have classically been quantified in behavioral paradigms validated by pharmacology^49^. Our framework, beyond identifying defensive states in standard behavioral assays used to model fear and anxiety, demonstrates that context is a major determinant of defensive microstates that appear identical on the behavioral level but exhibit different cardiac components. In addition, our data reveal large differences between various defensive macrostates in contexts that explicitly provide safe environments (EPM and LDB) and a uniform macrostate elicited in contexts that do not (OF).

We show that analyses of biologically relevant readouts of different modalities allow differentiation of behaviorally similar group differences. Ideally, unsupervised clustering algorithms should be used to identify subgroups within a global ‘state space’ as a basis for identifying new biomarkers for normal and pathological state dynamics. In taking a step back with a stringent data-driven approach, we provide a framework for the characterization of multimodal, integrated defensive states, thereby reopening a pathway toward translational research across species and from normal to maladapted fear and anxiety.

## Methods

### Animals

Two to 6-months old C57BL/6 male wild type and transgenic mice were bred in an in-house animal facility. *Slc17a6*^*tm2(cre)Lowl*^ (*Vglut2-ires-Cre*) knock-in mice were initially obtained from Jackson Laboratory (stock number 028863). *Chx10-ires-Cre* mice were bred from parents kindly provided by O. Kiehn (University of Copenhagen)^50^. *Slc17a6*^*tm1.1(flpo)Hze*^ (*Vglut2-ires2-FLPo-D*) knock-in mice, obtained from Jackson Laboratory (stock 030212) and *Chx10*^*Cre*^ mice were cross-bred in-house. All mice were individually housed at constant temperature 22 ± 1.5 °C and humidity of min. Fifty-five percent of 12 h light/12 h dark cycle and experiments were performed during the light cycle. Food and water were available ad libitum. All animal procedures were approved by the local veterinary authorities and animal experimentation ethics committee (Regierung von Unterfranken, authorization 2532-2-509). Present reporting follows ARRIVE guidelines (Animal Research: Reporting of In Vivo Experiments)^51^. No statistical methods were used to predetermine sample sizes but our sample sizes were estimated based on the previous studies using similar experimental designs^24,33^. Mice were randomly assigned to experimental and control groups.

### Stereotactic surgeries

AAVs were used as recombinant viral vectors to deliver genetic constructs of interest. Viruses for optogenetic experiments were produced in an in-house viral production facility from plasmids ordered from Addgene (pAAV-hSyn-flex-ReaChR-Citrine, 50955; pAAV-hSyn-DIO-mCherry, 50459) or ordered ready-made from Addgene (pAAV-FLEX-ArchT-tdTomato, 28305; pAAV-nEF-Coff/Fon-ChRmine-oScarlet, 137160). Mice were anesthetized with isoflurane (Harvard Apparatus, Iso-Vet Chanelle) in O_2_-enriched air (induction: 4%, maintenance: 1.5–2%). For systemic intra-operative analgesia, buprenorphine (0.05–0.1 mg kg^−1^; Buprenovet, Bayer) was administered subcutaneously 20 min before the start of the surgery. During the surgery, body temperature was kept stable by keeping the animal on a heating pad. Mice were fixed in a stereotactic frame (Model 1900, Kopf) and local analgesia was achieved by injecting ropivacaine under the scalp (8 mg kg^−1^; Naropin, AstraZeneca), after which a midline incision was performed. For viral injections targeting the vlPAG, craniotomies (0.4 mm diameter) were drilled 4.6 mm caudally and ±0.5 mm laterally from bregma. A calibrated glass pipette (calibrated micropipette 1–5 µl; Drummond Scientific) filled with the appropriate virus was slowly lowered to the target depth of 2.9 mm below bregma. A volume of 70–100 nl was injected at a speed of 25 nl min^−1^ with a pressure injector (PDES-02DX, NPI electronic). After injection, the capillary was held in place for 10 min, before being slowly withdrawn. The wound was sutured and treated with antiseptics. To ensure postoperative recovery analgesia, meloxicam (5 mg kg^−1^ every 12–24 h; Metacam, Boehringer Ingelheim) was administered subcutaneously after the operation was completed. For optogenetic light delivery to the target brain region, custom-built optical fiber stubs (ceramic ferrules, Thorlabs; multimode fiber 0,39 NA 200 µm, Thorlabs) were implanted in a second surgery. Salp was opened by removing a skin patch of 0.7 × 0.7 mm and skull was cleaned and carefully dried. Craniotomies were performed 4.6 mm caudally and ±1.6 mm laterally to bregma. Fiber stubs were inserted in a 20° angle to a depth of −2.1 mm from brain surface and secured with cyanoacrylate glue.

### Measurement of the ECG

To measure HR parameters, all animals were implanted with ECG electrodes. For this purpose, three PFA-coated wires (7SS-1T, Science Products) were soldered onto micro connectors (A79108, Omnetics; MSA components). Two wires were used to record the ECG signal differentially, the third was used as a reference. During the surgery, two small incisions were made on the upper right and lower left torso, at the ends of an imaginary diagonal centered on the heart. Wires were threaded through a blunt feeding needle in a subcutaneous tract carefully created from the skin incision on the front side of the mice towards the scalp opening. The distal ends of the wires were stripped of their insulation over 3 mm and sutured onto muscle tissue of the thorax. The skin was closed, disinfected and the connector was fixed on the skull.

### Optogenetics

After a recovery period of at least 1 week after surgery, animals were handled 4 d before the first recording session to habituate them to the connection procedure. For optical stimulation, a LED fiber light source was used (Ce:YAG optical head, Doric; 582 nm bandpass filter for ArchT and ChRmine and 612 nm bandpass filter for ReaChR). Stimulation protocols were created in Radiant (Plexon), and analog output from PlexBright (Plexon) was used to control stimulations’ intensity and patterns. For naïve testing of optogenetically-evoked effects, animals were placed into cylindric contexts (30 cm diameter), the floor being covered with HC bedding. *Vglut2*^*Cre*^ and Vglut2FlpO^*FlpO*^/*Chx10*^*Cre*^ animals were stimulated five times for 10 s at either low (0.3–0.5 mW, constant) or high intensities (3.5 mW, 10 Hz, 20 Hz or constant) in two separate sessions. *Chx10*^*Cre*^ animals similarly received five times 10 s stimulations at 30 Hz stimulation frequency (7.2 mW). For short stimulations (1.1 and 2 s alternating, 12 stimulations in total, stimulation settings see above) of *Chx10*^*Cre*^, subjects were placed into an OF context (50 × 50 cm) without bedding. For loss-of-function experiments, *Chx10*^*Cre*^ animals were conditioned with the Conditioned flight paradigm (see below). After 2 d of conditioning, animals were placed back in the conditioning context for 15 min and stimulated four times for 20 s (12 mW, constant). Data collection of optogenetic experiments was not performed blind to the conditions of the experiments.

### Histology and microscopy

Mice were anesthesized with a mixture of Ketamin (100 mg kg^−1^) and Xylazine (10 mg kg^−1^), injected intraperitoneally, and transcardially perfused with 1 x PBS and 4% paraformaldehyde (PFA) for 5 min each. Brains were dissected and postfixated overnight in PFA at 4 °C. Samples were washed, embedded in 6% agarose cubes and cut into 60 µm coronal sections using a vibratome (Leica VT1200). Sections of *Vglut2*^*FlpO*^/*Chx10*^*Cre*^ animals were immunohistochemically stained (primary antibody: rabbit anti-RFP, Rockland, 600-401-379, dilution 1:2,000; secondary antibody: donkey anti-rabbit Cy3, Jackson Immuno Research, 711-165-152, dilution 1:1,800). Sections were incubated in DAPI (4′,6-diamidino-2-phenylindole), mounted onto object slides and embedded with custom-made glycerol-based medium (Fluorostab) before imaging with a fluorescence microscope (AxioImager 2, Zeiss). Mice with no or unilateral expression of the fluorescent tag or with fiber placement outside the PAG were excluded from the analysis.

### Behavior recordings

Behavioral experiments were conducted in two different sound-attenuated chambers (length: 100 cm, width: 80 cm and height: 116 cm) lit from above by an adjustable circular LED lamp (LED-240; Proxistar). One chamber was covered with white insulating foam, brightly lit (350 Lux), and contained a Petri dish filled with 70% ethanol (chamber E), while the other was covered with black insulating foam, dimly lit (130 Lux), and contained a Petri dish filled with 1% acetic acid (chamber A). For the different paradigms, the appropriate contexts were placed in the center of either of the chambers and were cleaned with the corresponding liquid before each recording (either ethanol or acetic acid). Context temperature was kept at 22.5 ± 1 °C. Different paradigms were used to capture as many different states as possible. Mice with entangled cables that prevented free movement were excluded from further analysis.

#### HC

Animals were recorded in their HC and left unperturbed for 15 min.

#### OF

The apparatus consisted of a 50 × 50 × 50 cm white box. Animals were placed inside the middle of the box and left to freely explore the environment for 15 min.

#### EPM

The apparatus consisted of two open and two enclosed arms (8 cm width, 28 cm arm length and 28 cm wall height), elevated 25 cm above the chamber floor. Animals were placed in the crossing area at the intersection of the four arms, facing an open arm, and left to freely explore for 15 min.

#### LDB

The context box consisted of two compartments of identical dimensions (30 × 15 cm) that communicated via a 5 cm opening. One side was made of white material, with LED strips fixed near the upper rim providing illumination restricted to that compartment (350 LUX), while the other was black and void of any light source (50 LUX). Animals were habituated to dim light 15 min before the experiment.

#### Conditioned flight paradigm

The conditioned flight paradigm^24^ is a Pavlovian fear conditioning paradigm in which an SCS is used as the conditioning stimulus (CS) that is paired with the unconditioned stimulus, a shock. The SCS consists of a 10-s pure tone period (7.5 kHz, 75 dB, 500 ms beeps and 1 Hz) followed by a 10-s white noise period (1–20 kHz, 75 dB, 500 ms bursts and 1 Hz). On the first day (pre-exposure), the SCS alone is presented four times in a white cylinder (27 cm diameter). The conditioning was then performed on two consecutive days, with five SCS/US pairings on each day after a 3-min baseline period (pseudorandomized inter-stimulus interval (ITI): 170–230 s). The conditioning context was a red transparent square box (30 × 30 cm) with a grid floor through which footshocks were delivered. For each SCS/US pairing, a 1-s footshock (0.9 mA; Model 2,100 Isolated Pulse Stimulator, A-M System) immediately followed the last white noise burst. On the fourth day, animals underwent cue retrieval in a transparent cylinder (30 cm diameter), to receive 16 SCS presentations, without US pairings (pseudorandomized ITI: 80–140 s). One group of mice was placed into their HC while replaying the SCS tones instead a new context. A subset of mice underwent an additional day of recording and were placed back in the conditioning context for 15 min on the fifth day of the protocol (context retrieval).

Days 1 and 4 of the conditioned flight paradigm (pre-exposure and cue retrieval) and OF, EPM and LDB recordings were performed in context A (acetic acid smell, dim light), while recordings for days 2, 3 and optionally 5 of the conditioned flight paradigm (conditioning, context retrieval) were made in context E (ethanol smell, brighter light).

#### Retrieval in HC with long baseline

Animals were conditioned as described above. On the fourth day (retrieval), animals were placed in their HC and left unperturbed for 40 min. The pure tone component (7.5 kHz, 75 dB, 500 ms beeps, 1 Hz) of the SCS was played for 2 min. The total recording time was 50 min.

### Recording system

The overall recording system consisted of several main elements. An acquisition system (Plexon, Omniplex system) recorded analog and digital signals and was coupled with CinePlex Studio (Plexon) for synchronized top RGB camera recordings (Pike Camera F-032C, Allied Vision, Campden Instruments). The Radiant software was used to create optogenetics stimulation protocols and control the global timing of experiments via PlexBright analog and digital outputs. An RZ6 multi-processor (Tucker-Davis Technologies) was used to deliver acoustic stimulations via a multi-field magnetic speaker (MF1, Tucker-Davis Technologies), control the shocks delivered by the stimulus isolator and overall provide online processing and synchronization via a MATLAB/ActiveX/RPvdsEx interplay (MATLAB2019b, The MathWorks; RPvdsEx, Tucker-Davis Technologies). In particular, a fast initial train of TTLs followed by a 1 Hz signal was generated and broadcasted to the different systems for offline alignment. Temperature data were acquired with a long wavelength infrared camera (A655sc, FLIR), via FLIR’s SDK within MATLAB. A custom GUI was used for pre-recording calibration and focus, and to visualize the movie in real time. Recordings were triggered by MATLAB after the running signal was broadcasted by PlexBright and received by the RZ6, and similarly stopped. Thermal data was directly saved into a.seq file and MATLAB was periodically saving the current number of acquired frames which allowed for offline synchronization. ECG data were acquired at 5 kHz via an amplifier (DPA-2FX, npi) connected to the OmniPlex system. Depending on the quality of the signal for the two electrodes, signal was saved differentially or from single electrodes.

### HR analyses

Detection and extraction of heartbeats from the raw ECGs were performed within a MATLAB GUI (custom code, Supplementary Video 6). Briefly, the raw signal was read from the .pl2 files using Plexon’s SDK. When needed, bandpass filtering was applied after adjusting the frequencies. The resulting signal was then raised to the fourth power to increase separation and smoothed with a Gaussian filter. A threshold was then defined to extract putative heartbeats. After the timestamps were obtained from the modified signal, putative heartbeat waveforms were extracted from the signal. A divergent template-matching and interbeat interval confidence scoring algorithm were used to pretreat the results, with high specificity. Uncertain ranges were left to be manually fixed by the experimenter. If that was impossible or there was any doubt because of a bad signal-to-noise ratio (for example, contamination by electromyogram), the concerned ranges were marked and excluded from further analyses. The R peaks were then extracted from each waveform and saved for further processing. HR analysis was performed blind to the conditions of the experiments. Mice with ECG recordings that did not allow to extract R peaks reliably (for example, because of contamination by muscular potentials) were excluded from the analysis.

#### HR and wavelets

HR was processed from the R peaks using a sliding window of 0.6 s ending at each peak and resampled from these R peaks-based times to a fixed sampling rate. Continuous 1D wavelet transform (MATLAB wavelets toolbox, The MathWorks) was used to extract the frequency band of interest (0.4–0.8 Hz), which corresponds to what has been hypothesized to be the mouse equivalent of the LF band of HRV and the 0.1 Hz Mayer waves in humans^29^, closely related to baroreflex function.

#### Theoretical maximum HR (‘ceiling’)

The local theoretical maximum HR curve was obtained from a resampled (4 Hz) and median filtered HR (sliding window of four samples, centered around each sample) for the whole recording, by first extracting local maxima (sliding window, with more samples in the backward direction), and then smoothing with a sliding quadratic linear regression (‘smoothdata’ function in MATLAB, with ‘loess’ as method).

### Mouse tracking and behavior scoring

Raw top-view movies were processed with custom MATLAB code. For RGB movies, the 80th percentile of a manually or automatically selected set of frames was used as background that was subtracted from all the frames. Then both for RGB and thermal movies, a threshold was manually selected on a GUI, and the resulting binary mask underwent a series of simple treatments: morphological closing, removal of small objects, another morphological closing and finally morphological opening. The values for the different parameters were set to accommodate the different conditions. For each frame, mice contour and center of gravity were obtained from the resulting mask and saved. A calibration (px_movie_ / cm_real object_ ratio) was also obtained from a manually drawn segment and the corresponding length of the object in cm, for later normalization. In the following analysis steps, speed and mouse position were derived from the center of gravity coordinates, which were slightly smoothed with a median filter. In addition, to capture general activity, even in the absence of locomotion, a motion measure was used: it is computed as percentage of pixel change in the mouse masks from one frame to the next (nonoverlapping pixels/total pixel count). Several body parts (snout, ears, front and hind paws and tail) were also tracked with python-code-based DeepLabCut^52,53^. Briefly, a Resnet-152 network was iteratively trained and refined on ~1,350 frames to be as performant as possible in all our various recording conditions and in particular yield accurate tail tracking (*cf* thermal data extraction). To not sacrifice any accuracy, only coordinates with a score ≥0.99 were included, and no interpolation was applied. A semi-automated threshold-based GUI was used to annotate the following behaviors: rearing, grooming, stretch-attend posture, head dips, immobility, fast locomotion and so-called area-bound (not any of the other defined behaviors, in particular, no immobility and no locomotion). Briefly, for each behavior, a global score was obtained from relevant position information, body parts’ angles/distances/speed and thresholded with their respective hard-coded thresholds. The behavioral bouts resulting from that initial detection were displayed in a GUI together with the original movie and the scores. The thresholds could then be dragged manually, updating the detected events plots, to get the best possible detection. Events were then checked and adjusted manually when needed within the same GUI, and occasional periods of obstruction (for example, cable between the camera and the mouse) were marked for later exclusion. In the specific case of the LDB, because the RGB camera was not able to capture the mouse’s activity in the dark side, thermal movies were used for behavioral detection. To this end, thermal movies were re-exported with a black-and-white color map, after inverting the intensities and adjusting the contrast, so that the resulting frames resemble their RGB counterparts. A DeepLabCut network was derived from our main network and refined with those new movies (tail points were discarded because of their changing nature on thermal movies). The tracked body parts were then used as previously mentioned to detect behaviors. Mouse tracking was performed blind to the conditions of the experiments.

#### Spatial analyses

Areas in the different contexts were derived from the contours drawn during tracking, completed if necessary by manual delineation (LDB). In the case of the OF, the center corresponds to the middle square obtained when dividing the OF into a 5 × 5 grid, the corners correspond to the corners of that grid, and the corridors are the squares between two corners. Raw heat maps were generated from the appropriate data and the tracking information using FMA Toolbox (http://fmatoolbox.sourceforge.net/) with 250 bins and no smoothing. A postprocessing 2D smoothing was applied, ignoring bins without any occupancy to prevent ‘edge’ effects. Corresponding quantification was performed independently from the heat maps, by simply extracting from the raw data the time bins for each area and processing it accordingly (for example, averaging). For transition between areas, typical motifs were identified into the list of sequential areas explored by each mouse and sorted to keep the episodes starting from low speed and without immobility between starting area and target area, except for the OF for which the transition was used as synchronizing event for the PSTH because of the scarcity of events matching the criteria.

### Thermal data extraction

Following acquisition, the large.seq files were read in MATLAB using FLIR SDK (FLIR) and converted to .mj2 files after limiting the values range to 15–50 °C and conversion to 8 bits. The thermal movie was subsequently processed using the same GUI as for the RGB top camera, and mouse position, contour and motion were saved in the same file. Using either synchronization data that were saved online or fitting of the RGB and thermal motion curves, the initial shift and progressive drift between RGB and thermal cameras were determined, and the timestamps were adjusted accordingly to align the two cameras temporally. In addition, a Procrustes analysis on the mouse’s global track from both cameras was used to determine the transformation linking the coordinates of both cameras. Checkerboard calibration was performed for later recordings, but Procrustes transform was used over the entirety of the dataset for uniformity. The transformation was then applied to the coordinates of the body parts coming from the RGB camera with DeepLabCut, to obtain their equivalent on the thermal camera, the five points tracked for the tail defining four segments. Although our DeepLabCut network yielded excellent accuracy, and even though the transfer from RGB to the thermal camera was reliable, the actual tail width in some large contexts could represent as little as 2 px in the movies. Therefore, the coordinates of a rectangle extending 3 mm on each side of the segment were computed (using the px/mm calibration) for each segment, and the temperature was retrieved as the highest value from the corresponding mask. Because of the 2D nature of the tracking of a 3D object and of the coordinates transfer, the temperature extraction was subjected to an inherent minimum level of noise (vertical tail, not the same length visible, etc.). To correct for it, the data were postprocessed to get rid of some artifacts: first, a rank-order filter (90th percentile, 300 samples; Arash Salarian (2021), Rank-Order Filter; https://www.mathworks.com/matlabcentral/fileexchange/22111-rank-order-filter, MATLAB Central File Exchange) was applied to the signal and a threshold was used to localize artifacts. In a similar manner, moving averages obtained from different window sizes were substracted from the signal and the results thresholded to identify artefacts. If the resulting ranges were smaller than 15 s, missing data were linearly interpolated; otherwise, the values were set to NaN to be discarded from further analyses, and the results were saved.

### Rigidity model and statistics

Although we show that rigidity also affects the HR component of other microstates (for example, rearing-associated tachycardia), it is most easily accessed through immobility-associated bradycardia, a microstate with particular relevance to threat-related reactions, and as a consequence, one that is well represented throughout our various conditions. We thus used variations in the amplitude of those cardiac changes as a proxy for rigidity.

Because the peak amplitude of immobility-associated bradycardia (Bradycardia_IB_ measured as beats per minute) depends both on such within-session rigidity and on bout duration (Duration_IB_ in seconds) we computed coefficients for both factors (SessionCoefficient *A*_s_ and BoutDurationCoefficient *B*_d_, respectively, both in beats per minute per second, strictly negative and bounded within physiological ranges). This can be formalized as

1 $${\mathrm{Bradycardia}}_{{\mathrm{IB}}} = A_{\mathrm{s}} \times {{{\mathrm{t}}}} + B_{\mathrm{d}} \times {\mathrm{Duration}}_{\mathrm{IB}}\,({\mathrm{bpm}})$$

*A*_s_ captures the slow decrease in rigidity, which can be described as a relaxation process resulting in the decreased influence of rigidity on bradycardic responses over session time (*t*). Taking this into account, we introduce rigidity as a gain factor, scaling the range (Bradycardia_hmax_) of an individual’s possible HR changes. Thus, it constitutes an important parameter defining the momentary ‘state space’ of the cardiac system. In our rubber band analogy, it is the maximal amplitude of deflection. Rigidity works against this as the preload of the system (or the stretching force on the rubber band), thereby constricting deflection amplitude, or in other words, contracting the state space. Therefore, we define

2 $${\mathrm{Bradycardia}}_{{\mathrm{IB}}} = {\mathrm{Rigidity}}\left( {{t}} \right) \times {\mathrm{Bradycardia}}_{\mathrm{{hmax}}} + B_{\mathrm{d}} \times {\mathrm{Duration}}_{{\mathrm{IB}}}\;\left( {\mathrm{bpm}} \right)$$

In relation to the SessionCoefficient, this means that

3 $$\begin{array}{l}A_{\mathrm{s}} \times {{t}} + B_{\mathrm{d}} \times {\mathrm{Duration}}_{{\mathrm{IB}}} =\\ {\mathrm{Rigidity}}\left( {{t}} \right) \times {\mathrm{Bradycardia}}_{{\mathrm{hmax}}} + B_{\mathrm{d}} \times {\mathrm{Duration}}_{{\mathrm{IB}}}\;\left( {\mathrm{{bpm}}} \right)\end{array}$$

resulting in a dimension-less definition of

4 $${\mathrm{Rigidity}}({{t}}) = \frac{{{\mathrm{Bradycardia}}_{{\mathrm{IB}}}-B_{\mathrm{d}} \times {\mathrm{Duration}}_{{\mathrm{IB}}}}}{{{\mathrm{Bradycardia}}_{{\mathrm{hmax}}}}}\;\left( {\mathrm{no}}\,{\mathrm{unit}} \right)$$

or

5 $${{{\mathrm{Rigidity}}}}\,\left( {{t}} \right) = \frac{{{t}}}{{{{{\mathrm{Bradycardia}}}}_{{{{\mathrm{hmax}}}}}}} \times {{{{A}}}}_{{{{{\mathrm{s}}}}}}\;\left( {\mathrm{no}}\,{\mathrm{unit}} \right)$$

We found immobility-associated bradycardia to depend on both an intrinsic characteristic, namely immobility bout duration, and one general modulatory influence (herein termed rigidity).

Both to better characterize such defensive response (immobility-associated bradycardia) but also to quantify rigidity, both factors were integrated into a simple model that describes immobility-associated bradycardia’s amplitude in function of time (main contributor to slow rigidity changes) and bout duration.

Practically, to prevent skewing from, for example, individual mice presenting numerous episodes, or single outlier episodes, a fitting was not performed on the pooled individual values per single episode. Instead, average amplitudes of bradycardia were processed for overlapping ranges of immobility bout durations spanning their spread, and at the same time, overlapping time ranges mapping the recording session (for example, retrieving the minimum value on an average PSTH of HR-to-ceiling for immobility episodes lasting from 2 to 2.5 s and occurring between 0 and 60 s would give one data point, with a given bradycardia value for 2.25 s bout duration and 30 s session time). The resulting values were fitted using a polynomial equation; robust fitting without normalization was used (MATLAB curve fitting toolbox, The MathWorks). *R*² values and statistical comparisons were obtained for the simple linear model from equation (1). *A*_s_ and *B*_d_ values are strictly negative as they capture rigidity relaxation, and the corresponding linear equation is valid only for a certain range of session and bout durations where the relationship between bradycardia and time variables has not reached an asymptotic level. This is the case for our conditions, but a more robust model could alter some terms to introduce an exponential decay.

It comes from equation (5) that rigidity at a given time is directly proportional to the SessionCoefficient *A*_s_, and comparing *A*_s_ between different contexts would therefore be equivalent to comparing their rigidity. However, we chose to display a numerical value for rigidity on the corresponding plots to give an idea about its scale. To this end, we estimated Bradycardia_hmax_ based on the minimum and maximum HR values observed in mice under relevant conditions (awake, no physiological alterations). This indicated a range of 450–800 bpm (refs. ^21,54–59^), and therefore a Bradycardia_hmax_ of 350 bpm. Because equation (5) defines rigidity at a given time point, we chose to compare it at *t* = 600 s. It follows that

6 $${{{\mathrm{Estimated}}\, {\mathrm{rigidity}}}}\left( {600{{{{{\mathrm{s}}}}}}} \right) = \frac{{600}}{{350}} \times {{{{A}}}}_{{{{\mathrm{s}}}}}\;\left( {\mathrm{no}}\,{\mathrm{unit}} \right)$$

We used an *F*-statistic (equation (7)) to compare different conditions (for example, open versus closed arm). The fits of the corresponding separate surfaces were compared against the fit of a single surface to fit both conditions (for example, the whole EPM in this case). A *P* < 0.05 was considered statistically significant, which implicated the datasets were so different that they were best described by two different models (adapted from ref. ^60^)

7 $$F = \frac{{\begin{array}{l}({\mathrm{SScombined}} - ({\mathrm{SScondition}}1 + {\mathrm{SScondition2}}))/\\({\mathrm{DFcombined}} - ({\mathrm{DFcondition}}1 + {\mathrm{DFcondition}}2))\end{array}}}{{({\mathrm{SScondition}}1 + {\mathrm{SScondition}}2)/\left( {{\mathrm{DFcondition}}1 + {\mathrm{DFcondition}}2} \right)}}$$

Here SS is sum of squares error and DF is degrees of freedom.

### K-means clustering

The BIC was used to determine the optimal number of clusters (Mclust package^61^ in R^62^) on per-mouse averages for conditioning day 2 (speed, motion, HR-to-ceiling, LF amplitude and thermographically recorded tail temperature were used). K-means clustering with the predetermined number of clusters was performed in Matlab (squared Euclidean distance, 1,000 replicates).

### UMAP embedding

The different readouts were resampled to a common time vector (4 Hz) in MATLAB and exported to a csv file to be embedded with UMAP^39^ (0.5.0) using Euclidean distance, 20 neighbors and a minimum distance of 0.3 in either two or three dimensions. The results of the embedding were exported as xls files and read back into MATLAB where the reduced data were matched with the original readouts and metadata.

For the across-day embedding, 250 UMAP analyses were performed with the relevant data (HR-to-ceiling, immobility bout duration, immobility-associated bradycardia and LF amplitude during locomotion) from all included days each time. The Euclidean distances presented in the matrices are the resulting average of the distances for the 250 runs. Color coding for the EPM subareas was added post hoc by using the metadata.

Embedding for conditioning day 2 was used only for visualization purposes and performed on speed, motion, HR-to-ceiling, LF amplitude and thermographically recorded tail temperature.

### Specific analyses

For any PSTHs, the appropriate data were extracted around the synchronizing events and resampled to fixed timestamps to allow for cross-trial and cross-animal averaging. 3D representations were built from PSTHs processed over overlapping time windows (60 s width, 50 s overlap).

### Statistics

Normality was checked using Lilliefors test for each set of data, and homoscedasticity was tested with Brown-Forsythe test. When only two sets of data were compared, and if the hypothesis of normality was true, Student’s *t*-test was used, otherwise, Wilcoxon signed-rank test or Mann–Whitney *U* test was used. When more than two sets of data were compared, a one-way ANOVA test was used if the hypotheses of equal variance and normality were true for all, otherwise, a Kruskal–Wallis test was used in both cases followed by an appropriate post hoc test with Bonferroni correction. A mixed model ANOVA was used for the analysis of the behaviors x time interactions supporting the results of Fig. 3a and presented in Extended Data Fig. 3c,d. For the comparison of the intertrial values as presented in Fig. 6, two-way RM ANOVA followed by Sidak’s multiple comparisons test was performed in Prism 7.05 (GraphPad).

### Reporting summary

Further information on research design is available in the Nature Portfolio Reporting Summary linked to this article.

## Online content

Any methods, additional references, Nature Portfolio reporting summaries, source data, extended data, supplementary information, acknowledgements, peer review information; details of author contributions and competing interests; and statements of data and code availability are available at 10.1038/s41593-022-01252-w.

## Supplementary information

Supplementary Information Supplementary Table 1.

Reporting Summary

Supplementary Video 1 Representative example videos of spontaneous and evoked freezing microstates as well as rearing.

Supplementary Video 2 Optical activation of *VGlut2*^+^ vlPAG neurons of a naïve mouse with low- and high-intensity stimulation.

Supplementary Video 3 Optical activation of *Chx10*^+^ vlPAG neurons of a naïve mouse with high-intensity stimulation.

Supplementary Video 4 Optical inhibition of *Chx10*^+^ vlPAG neurons of a mouse that underwent fear conditioning (conditioned flight paradigm) and is re-exposed to the conditioning context during optogenetic experiments.

Supplementary Video 5 Optical activation of *VGlut2*^+^/*Chx10*^−^ vlPAG neurons of a naïve mouse with low- and high-intensity stimulation.

Supplementary Video 6 Introduction into custom-written MATLAB-based Heart_Rate_Extraction_Tool used for the R-Peak detection of heart beats.

## Data Availability

Data supporting the findings of this study are available from the corresponding author upon reasonable request.

## Code availability

The custom code used to preprocess ECGs and contour tracking is available at https://github.com/DefenseCircuitsLab/. Other code used in this study is available from the corresponding author upon reasonable request.
