# Supplementary material for: Integrated cardio-behavioral responses to threat define defensive states
Source: Nat Neurosci. 2023 Feb 9;26(3):447–57. doi: 10.1038/s41593-022-01252-w (PMC9991919; doi:10.1038/s41593-022-01252-w)
Supplement: Supplementary file 1 — Supplementary Table 1. [file 41593_2022_1252_MOESM1_ESM.pdf]

nature neuroscience

Article

https://doi.org/10.1038/s41593-022-01252-w

Integrated cardio-behavioral responses to threat define defensive states

In the format provided by the authors and unedited

Supplementary Table 1

Figure	Normality test	Pairwise test	Comparison	Tail	p-value	Group analysis	F-statistic (or Chi-square)	p-value
2b	passed	unpaired Student's t-test	HR vs HR-to-ceiling	two-tailed	1.2291e-07			
3a	failed	post-hoc with Bonferroni correction	Behaviours	two-tailed	cf matrix	Kruskal-Wallis	(5,164) = 118.7625	5.7388e-24
3b	failed	post-hoc with Bonferroni correction	Contexts	two-tailed	cf matrix	Kruskal-Wallis	F(8,145) = 81.8844	2.0398e-14
4a	passed	post-hoc with Bonferroni correction	EPM subareas	two-tailed	Open arm vs closed arm: 5.57469e-05<br>Center vs closed arm: 0.02329	one-way ANOVA	F(2, 60) = 10.9428	8.8782e-05
4b	passed	post-hoc with Bonferroni correction	LDB subareas	two-tailed	Light vs corridor: 0.033799<br>Light vs dark: 0.0092517	one-way ANOVA	F(2, 42) = 5.7107	0.0064004
4c	passed	-	OF subareas	-	-	one-way ANOVA	F(2, 66) = 0.21763	0.805
4j	passed	post-hoc with Bonferroni correction	EPM subareas	two-tailed	Open arm vs closed arm: 2.1636e-05<br>Center vs closed arm: 0.012626	one-way ANOVA	F(2, 56) = 12.377	3.5372e-05
4k	passed	-	LDB subareas	-	-	one-way ANOVA	F(2, 30) = 2.3881	0.10904
4l	passed	-	OF subareas	-	-	one-way ANOVA	F(2, 58) = 0.23383	0.79224
4d top	passed	unpaired Student's t-test	Closed to open vs open to closed	two-tailed	1.3966e-10			
4d bottom	failed	Mann–Whitney	Closed to open vs open to closed	two-tailed	3.9446e-07			
4e top	passed	unpaired Student's t-test	Light to dark vs dark to light	two-tailed	1.6518e-04			
4e bottom	passed	unpaired Student's t-test	Light to dark vs dark to light	two-tailed	0.7214			
4f top	failed	Mann–Whitney	Inner to outer vs outer to inner	two-tailed	0.3345			
4f bottom	failed	Mann–Whitney	Inner to outer vs outer to inner	two-tailed	0.0022586			
4g	passed (no homoscedasticity)	post-hoc with Bonferroni correction	EPM subareas	two-tailed	Open arm vs center: 0.0995780<br>Open arm vs closed arm: 4.1001e-07	Kruskal-Wallis	(2,60) = 28.1091	7.8737e-07

4h	passed (no homoscedasticity)	post-hoc with Bonferroni correction	LDB subareas	two-tailed	Light vs corridor: 2.3937e-08<br>Light vs dark: 0.0084048<br>Dark vs corridor: 0.016298	Kruskal-Wallis	(2,42) = 33.2947	5.8905e-08
4i	failed	post-hoc with Bonferroni correction	OF subareas	two-tailed	Center vs corners: 2.8572e-04<br>Center vs inner ring: 0.0011572	Kruskal-Wallis	(2,66) = 18.6355	8.9816e-05
4n	-	-	Closed vs open arm	one-tailed		F test	(3,419) = 15.9772<br>(with p = 1-df(F))	0
5g	passed	post-hoc with Sidak correction	Controls vs ArchT (immobility during inhibition)	two-tailed	Trial 1: <0.0001<br>Trial 2: 0.0134<br>Trial 3: 0.0314<br>Trial 4: 0.1126	RM ANOVA	(1, 7) = 15.62	0.0055
6f	-	-	Cluster 1 vs cluster 2	one-tailed		F test	(3,916) = 38.4872<br>(with p = 1-df(F))	7.4734e-10
Ext. Data 2a	failed	Mann–Whitney	HR vs HR-to-ceiling	two-tailed	8.8985e-04			
Ext. Data 2b	passed	unpaired Student's t-test	HR vs HR-to-ceiling	two-tailed	0.0017082			
Ext. Data 2g top	passed	paired Student's t-test	Pre-CS vs pure tone	two-tailed	0.86001			
Ext. Data 2h top	passed	paired Student's t-test	Pre-CS vs pure tone	two-tailed	0.0042966			
Ext. Data 2g bottom	failed	Wilcoxon signed-rank	Pre-CS vs pure tone	two-tailed	0.81408			
Ext. Data 2h bottom	passed	paired Student's t-test	Pre-CS vs pure tone	two-tailed	0.0041187			
Ext. Data 3a	passed	post-hoc with Bonferroni correction	Behaviours	two-tailed	cf matrix	one-way ANOVA	F(5,164) = 8.2619	5.5314e-07
Ext. Data 3b	failed	post-hoc with Bonferroni correction	Behaviours	two-tailed	cf matrix	Kruskal-Wallis	(5,164) = 74.1437	1.4037e-14
Ext. Data 3d	-	post-hoc with Tukey's test	Behaviours, time	two-tailed	-	RMANOVA (mixed model)	Behaviours: F (1.661, 202.1) = 58.43<br>Time: F (5, 165) = 42.95<br>Interaction: F (15, 365) = 14.08	All <0.0001
Ext. Data 3f	failed	post-hoc with Bonferroni correction	Contexts	two-tailed	cf matrix	Kruskal-Wallis	(8, 145) = 77.0866	7.5145e-13
Ext. Data 3g	failed	post-hoc with Bonferroni correction	Contexts	two-tailed	cf matrix	Kruskal-Wallis	(8, 145) = 77.0866	7.5145e-13
Ext. Data 3h	failed	post-hoc with Bonferroni correction	Contexts	two-tailed	cf matrix	Kruskal-Wallis	(8, 145) = 57.2016	1.6447e-09

Ext. Data 3i	passed (no homoscedasticity)	post-hoc with Bonferroni correction	Contexts	two-tailed	cf matrix	Kruskal-Wallis	F(8,145) = 60.9944	2.9733e-10
Ext. Data 4a left	passed	-	EPM subareas	-	-	one-way ANOVA	F(2, 60) = 0.048052	0.95312
Ext. Data 4a right	passed	post-hoc with Bonferroni correction	EPM subareas	two-tailed	Open arm vs closed arm: 0.02808	one-way ANOVA	F(2, 55) = 3.632	0.032997
Ext. Data 4b left	passed	-	LDB subareas	-	-	one-way ANOVA	F(2, 42) = 0.7631	0.47257
Ext. Data 4b right	passed	-	LDB subareas	-	-	one-way ANOVA	F(2, 30) = 2.0124	0.15131
Ext. Data 4c left	passed	-	OF subareas	-	-	one-way ANOVA	F(2, 66) = 0.022819	0.97745
Ext. Data 4c right	failed	-	OF subareas	-	-	Kruskal-Wallis	(2, 58) = 4.1024	0.12858
Ext. Data 4d bottom	failed	post-hoc with Bonferroni correction	EPM subareas	two-tailed	Open arm vs middle: 0.0046<br>Open arm vs closed arm: 0.0134<br>Closed arm vs middle: <0.001	Kruskal-Wallis	F(2, 54) = 36.1628	1.404e-08
Ext. Data 4e bottom	failed	post-hoc with Bonferroni correction	LDB subareas	two-tailed	Light vs dark: 0.0017<br>Corridor vs dark: <0.001	Kruskal-Wallis	F(2, 51) = 32.3964	9.2302e-08
Ext. Data 4f bottom	failed	post-hoc with Bonferroni correction	OF subareas	two-tailed	Center vs inner square: 0.0011<br>Center vs outer square: <0.001<br>Center vs corners: <0.001<br>Inner square vs outer square: 0.0046<br>Outer square vs corners: 0.0329	Kruskal-Wallis	F(3,72) = 51.1569	4.5296e-11
Ext. Data 4m	failed	post-hoc with Bonferroni correction	EPM subareas	two-tailed	Open arm vs closed arm: 0.022858	Kruskal-Wallis	(2, 60) = 7.5139	0.023355
Ext. Data 4n	passed (no homoscedasticity)	post-hoc with Bonferroni correction	LDB subareas	two-tailed	Corridor vs dark: 0.034221s	Kruskal-Wallis	(2, 42) = 7.6274	0.022066
Ext. Data 4o	passed	-	OF subareas	-	-	one-way ANOVA	F(2, 66) = 1.56	0.21779
Ext. Data 4p	passed	post-hoc with Bonferroni correction	EPM subareas	two-tailed	Open arm vs closed arm: 4.1732e-06	one-way ANOVA	F(2, 60) = 14.4068	7.7652e-06

					Center vs closed arm: 0.014911			
Ext. Data 4q	passed (no homoscedasticity)	post-hoc with Bonferroni correction	LDB subareas	two-tailed	Light vs corridor: 0.00033399<br>Light vs dark: 0.014955	Kruskal-Wallis	(2, 42) = 15.9567	0.0003428
Ext. Data 4r	passed	-	OF subareas	-	-	one-way ANOVA	F(2, 66) = 0.13698	0.87223
Ext. Data 4s	failed	post-hoc with Bonferroni correction	EPM subareas	two-tailed	Open arm vs center: 0.023973<br>Open arm vs closed arm: 0.0027776	Kruskal-Wallis	(2,34) = 12.51	0.0019208
Ext. Data 5e	passed	post-hoc with Sidak correction	Controls vs ArchT (motion during inhibition)	two-tailed	Trial 1: 0.0001<br>Trial 2: 0.0003<br>Trial 3: 0.0057<br>Trial 4: 0.0021	RM ANOVA	(1, 7) = 30.97	0.0008
Ext. Data 5e	passed	post-hoc with Sidak correction	Controls vs ArchT (HR-to-ceiling during inhibition)	two-tailed	Trial 1: 0.9426<br>Trial 2: 0.9983<br>Trial 3: 0.0015<br>Trial 4: 0.3389	RM ANOVA	(1, 7) = 7.808	0.0267
Ext. Data 5e	passed	-	Controls vs ArchT (intertrial immobility)	-	-	RM ANOVA	(1, 7) = 2.091	0.1914
