# Supplementary material for: Integrated cardio-behavioral responses to threat define defensive states
Source: Nat Neurosci. 2023 Feb 9;26(3):447–57. doi: 10.1038/s41593-022-01252-w (PMC9991919; doi:10.1038/s41593-022-01252-w)
Supplement: Supplementary file 2 — Reporting Summary [file 41593_2022_1252_MOESM2_ESM.pdf]

nature portfolio

Corresponding author(s): Philip Tovote (Tovote_P@ukw.de)

Last updated by author(s): Dec 20, 2022

Reporting Summary

Nature Portfolio wishes to improve the reproducibility of the work that we publish. This form provides structure for consistency and transparency in reporting. For further information on Nature Portfolio policies, see our Editorial Policies and the Editorial Policy Checklist.

Statistics

For all statistical analyses, confirm that the following items are present in the figure legend, table legend, main text, or Methods section.

n/a	Confirmed	
☐	☒	The exact sample size (*n*) for each experimental group/condition, given as a discrete number and unit of measurement
☐	☒	A statement on whether measurements were taken from distinct samples or whether the same sample was measured repeatedly
☐	☒	The statistical test(s) used AND whether they are one- or two-sided<br>*Only common tests should be described solely by name; describe more complex techniques in the Methods section.*
☒	☐	A description of all covariates tested
☐	☒	A description of any assumptions or corrections, such as tests of normality and adjustment for multiple comparisons
☐	☒	A full description of the statistical parameters including central tendency (e.g. means) or other basic estimates (e.g. regression coefficient) AND variation (e.g. standard deviation) or associated estimates of uncertainty (e.g. confidence intervals)
☐	☒	For null hypothesis testing, the test statistic (e.g. *F*, *t*, *r*) with confidence intervals, effect sizes, degrees of freedom and *P* value noted<br>*Give P values as exact values whenever suitable.*
☒	☐	For Bayesian analysis, information on the choice of priors and Markov chain Monte Carlo settings
☒	☐	For hierarchical and complex designs, identification of the appropriate level for tests and full reporting of outcomes
☒	☐	Estimates of effect sizes (e.g. Cohen's *d*, Pearson's *r*), indicating how they were calculated

Our web collection on statistics for biologists contains articles on many of the points above.

Software and code

Policy information about availability of computer code

Data collection	Analog signals and top RGB camera movies were recorded with Plexon's OmniPlex/PlexControl (v1.17.0, Plexon Inc.) and CinexPlex (v3.7.0.0, Plexon Inc.), respectively.<br>Thermal imaging data was acquired in Matlab 2019a (MathWorks) via the FLIR Atlas for Matlab SDK 5.0 (FLIR)<br>Control and synchronisation were achieved with the above-mentioned elements as well as Radiant (v2.2.0.19, Plexon Inc.) and Matlab interface for the RZ6 online processor with TDT ActiveX Controls (v7.3, Tucker-Davis technologies), also loading rcx files (RPvdsEx v95, Tucker-Davis technologies) , synchronisation signals being saved via RZ6/Matlab and PlexControl.<br>Microscopy data were acquired using Zeiss Zen Blue 2.5
Data analysis	Most analyses were peformed in Matlab 2019b (MathWorks)<br>The custom code used to preprocess ECGs and contour tracking is available at https://github.com/DefenseCircuitsLab/<br>Plexon analog and digital data was read into Matlab with the Plexon SDK (Plexon)<br>Thermal imaging data was read into Matlab using FLIR Atlas for Matlab SDK 5.0 (FLIR)<br>Some specific analyses were performed with Python code: DeepLabCut 2.1.8.1 (GPU version) was used to track bodyparts and UMAP 0.5 was used for dimensionality reduction<br>BIC analyses were performed in R (4.2.0), with the mclust5 package; Prism 7 was used for RM ANOVAs<br>Isolated functions from the The Freely Moving Animal toolbox (http://fmatoolbox.sourceforge.net/) were used, as well as the Rank-Order Filter function (https://www.mathworks.com/matlabcentral/fileexchange/22111-rank-order-filter)

For manuscripts utilizing custom algorithms or software that are central to the research but not yet described in published literature, software must be made available to editors and reviewers. We strongly encourage code deposition in a community repository (e.g. GitHub). See the Nature Portfolio guidelines for submitting code & software for further information.

Data

Policy information about availability of data

All manuscripts must include a data availability statement. This statement should provide the following information, where applicable:

- Accession codes, unique identifiers, or web links for publicly available datasets
- A description of any restrictions on data availability
- For clinical datasets or third party data, please ensure that the statement adheres to our policy

Data supporting the findings of this study are available from the corresponding author upon reasonable request.

Field-specific reporting

Please select the one below that is the best fit for your research. If you are not sure, read the appropriate sections before making your selection.

☒ Life sciences ☐ Behavioural & social sciences ☐ Ecological, evolutionary & environmental sciences

For a reference copy of the document with all sections, see nature.com/documents/nr-reporting-summary-flat.pdf

Life sciences study design

All studies must disclose on these points even when the disclosure is negative.

Sample size	Sample sizes were estimated based on previous studies using similar experimental designs (Tovote et al., Nature 2016, Fadok et al., Nature 2017).
Data exclusions	Non-readable data (e.g. obstructed ECG signal) were excluded from the analysis.<br>Animals were excluded based on histology data (no viral expression in ROI or fiber misplacement)
Replication	Experiments were replicated as indicated by n-Numbers and experimental design description in the Methods section.
Randomization	Assignment of animals to experimental groups was done randomly.
Blinding	Experimenters were blind during semi-automatic quantification of behavioural data. Blinding during acquisition was unnecessary because animals belonged to the same group (all basic behavioral/cardiac experiments) or were treated identically using automated stimulation protocols (optogenetics).

Reporting for specific materials, systems and methods

We require information from authors about some types of materials, experimental systems and methods used in many studies. Here, indicate whether each material, system or method listed is relevant to your study. If you are not sure if a list item applies to your research, read the appropriate section before selecting a response.

Materials & experimental systems

n/a	Involved in the study	
☐	☒	Antibodies
☒	☐	Eukaryotic cell lines
☒	☐	Palaeontology and archaeology
☐	☒	Animals and other organisms
☒	☐	Human research participants
☒	☐	Clinical data
☒	☐	Dual use research of concern

Methods

n/a	Involved in the study	
☒	☐	ChIP-seq
☒	☐	Flow cytometry
☒	☐	MRI-based neuroimaging

Animals and other organisms

Policy information about studies involving animals; ARRIVE guidelines recommended for reporting animal research

Laboratory animals	2- to 6-month old male C57BL/6 wild type, as well as transgenic mice (Slc17a6tm2(cre)Lowl, Chx10-ires-Cre) were used. In addition, Slc17a6tm1.1(flpo)Hze (Vglut2-ires2-FLPo-D) knock-in mice, obtained from Jackson Laboratory (stock number 030212) and Chx10-Cre mice were cross-bred in-house under standard holding conditions (see Methods).
Wild animals	No wild animals were used in this study.
Field-collected samples	No field collected samples were used in this study.

Ethics oversight	All animal procedures were approved by the local veterinary authorities and animal experimentation ethics committee (Regierung von Unterfranken, authorization 2532-2-509)

Note that full information on the approval of the study protocol must also be provided in the manuscript.
